# Human cytomegalovirus IE1 downregulates Hes1 in neural progenitor cells as a potential E3 ubiquitin ligase

**DOI:** 10.1371/journal.ppat.1006542

**Published:** 2017-07-27

**Authors:** Xi-Juan Liu, Bo Yang, Sheng-Nan Huang, Cong-Cong Wu, Xiao-Jun Li, Shuang Cheng, Xuan Jiang, Fei Hu, Ying-Zi Ming, Michael Nevels, William J. Britt, Simon Rayner, Qiyi Tang, Wen-Bo Zeng, Fei Zhao, Min-Hua Luo

**Affiliations:** 1 State Key Laboratory of Virology, CAS Center for Excellence in Brain Science and Intelligence Technology (CEBSIT), Wuhan Institute of Virology, Chinese Academy of Sciences, Wuhan, Hubei, China; 2 University of Chinese Academy of Sciences, Beijing, China; 3 Guangzhou Institute of Pediatrics, Guangzhou Women and Children Medical Center, Guangzhou, China; 4 Wuhan Brain Hospital, Ministry of Transportation, Wuhan, Hubei, China; 5 The Third Xiangya Hospital, South Central University, Changsha, Hunan, China; 6 School of Biology, Biomedical Sciences Research Complex, University of St Andrews, St Andrews, Fife, United Kingdom; 7 Department of Pediatrics, University of Alabama School of Medicine, Birmingham, Alabama, United States of America; 8 Department of Medical Genetics, Oslo University Hospital & University of Oslo, Oslo, Norway; 9 Department of Microbiology, Howard University College of Medicine, Howard University, Washington DC, United States of America; Blumburg Institute, UNITED STATES

## Abstract

Congenital human cytomegalovirus (HCMV) infection is the leading cause of neurological disabilities in children worldwide, but the mechanisms underlying these disorders are far from well-defined. HCMV infection has been shown to dysregulate the Notch signaling pathway in human neural progenitor cells (NPCs). As an important downstream effector of Notch signaling, the transcriptional regulator Hairy and Enhancer of Split 1 (Hes1) is essential for governing NPC fate and fetal brain development. In the present study, we report that HCMV infection downregulates Hes1 protein levels in infected NPCs. The HCMV 72-kDa immediate-early 1 protein (IE1) is involved in Hes1 degradation by assembling a ubiquitination complex and promoting Hes1 ubiquitination as a potential E3 ubiquitin ligase, followed by proteasomal degradation of Hes1. Sp100A, an important component of PML nuclear bodies, is identified to be another target of IE1-mediated ubiquitination. A C-terminal acidic region in IE1, spanning amino acids 451 to 475, is required for IE1/Hes1 physical interaction and IE1-mediated Hes1 ubiquitination, but is dispensable for IE1/Sp100A interaction and ubiquitination. Our study suggests a novel mechanism linking downregulation of Hes1 protein to neurodevelopmental disorders caused by HCMV infection. Our findings also complement the current knowledge of herpesviruses by identifying IE1 as the first potential HCMV-encoded E3 ubiquitin ligase.

## Author summary

Congenital human cytomegalovirus (HCMV) infection is the leading cause of neurological disabilities in children, but the underlying pathogenesis of this infection remains unclear. Hes1, an important effector of Notch signaling, governs the fate of neural progenitor cells (NPCs) and fetal brain development. Here we demonstrate that: (1) HCMV infection results in loss of Hes1 protein in NPCs; (2) the HCMV immediate-early 1 protein (IE1) mediates Hes1 protein downregulation through direct interaction, which requires amino acids 451–475; (3) IE1 assembles a Hes1 ubiquitination complex and mediates Hes1 ubiquitination; and (4) IE1 also assembles an Sp100A ubiquitination complex and mediates Sp100A ubiquitination, but does not require amino acids 451–475. These results suggest that HCMV IE1 is a potential E3 ubiquitin ligase. Downregulation of Hes1 by HCMV infection and IE1 implies a novel mechanism linking Hes1 depletion to virus-induced neuropathogenesis.

## Introduction

As a leading cause of birth defects, congenital human cytomegalovirus (HCMV) infection causes irreversible maldevelopment of the central nervous system (CNS) in newborns and children [1–4]. To understand how HCMV interferes with neurodevelopment, neural progenitor cells (NPCs) have been utilized as a clinically relevant model for investigation of the underlying mechanisms [5–10].

Proper self-renewal and differentiation of NPCs are fundamental to normal fetal brain development. Notch signaling is one of the best-characterized pathways governing NPC maintenance, proliferation and differentiation [11–13]. This regulatory role is achieved, at least partially, through essential downstream effectors such as the Hairy and Enhancer of Split (Hes) proteins, which belong to the repressor-type basic helix-loop-helix family [14, 15]. Hes1 is one of seven members in the Hes family, which play a crucial role in maintaining the undifferentiated and proliferative status of NPCs [16–18]. The auto-negative feedback regulation at the transcription level, the instability of the mRNA, and the rapid ubiquitination-dependent proteasomal degradation of the protein together result in the well-regulated Hes1 oscillation, which in turn fine-tunes the timing of NPC proliferation and differentiation and further controls the shape, size and integrity of brain structures [13, 19–21]. Studies in mice have shown that Hes1-deficient murine NPC neurospheres fail to expand, and Hes1 knockout accelerates neurogenesis from radial glial cells representing NPCs in mice [21–24]. These evidences imply that the dysregulation of Hes1 expression leads to abnormal NPC differentiation and proliferation, potentially contributing to fetal brain developmental disorders. We have previously reported that HCMV dysregulates Notch signaling by targeting Notch1, including its intracellular active domain (NICD), and the ligand Jag1 [5]. Moreover, as an important downstream effector in Notch signaling, the regulation of Hes1 expression in NPCs is disrupted by HCMV infection [25].

During HCMV infection of permissive cells, immediate early (IE) genes are the first to be expressed from the viral genome, and IE proteins can be detected as early as 2h post infection (hpi). IE proteins trigger viral early gene expression and, subsequently, viral genome replication and late gene expression. In addition, IE proteins also interact with multiple host factors to tune the cellular environment for initiation of viral replication. Thus, the synthesis of IE gene products is necessary for a full viral replication cycle [26, 27]. The most abundant and arguably most important HCMV IE gene products, termed IE1 and IE2, are encoded in the major IE transcription unit. The 72-kDa IE1 is a nuclear phosphoprotein and has been subject to extensive study [28]. The IE1 protein promotes the accumulation of IE2 gene products and synergizes with IE2 to activate viral early promoters [29–33], in part by antagonizing histone deacetylation, to facilitate virus replication [34, 35]. IE1 also affects host gene expression by activating or repressing transcription. For example, IE1 up-regulates the transcription of interleukin 6 (IL-6) and the genes activated by signal transducer and activator of transcription 1 (STAT1) [36]. Moreover, IE1 inhibits transactivation of p53-dependent downstream genes, disrupts transcription of STAT3-activated genes, and downregulates certain essential NPC markers such as the glial fibrillary acidic protein (GFAP) [37–40].

The recent study on the crystal structure of the IE1 ortholog from Macacine herpesvirus 3 (Rhesus cytomegalovirus) revealed striking similarities between IE1 and tripartite motif (TRIM) family proteins [41]. Many TRIM proteins possess E3 ubiquitin ligase activities [42], and E3 ubiquitin ligase activities have been described in several α- and γ-herpesvirus proteins, including ICP0 of herpes simplex virus type 1 (HSV-1), ORF61p of varicella-zoster virus (VZV), and replication and transcription activator (RTA), K3 and K5 of Kaposi’s sarcoma-associated herpesvirus (KSHV) [43–48]. However, to our knowledge, no E3 ubiquitin ligase has been identified in HCMV or any other β-herpesvirus so far.

In the present study, we demonstrated that HCMV infection downregulates Hes1 protein levels in infected human NPCs. Importantly, IE1 leads to Hes1 depletion by mediating Hes1 ubiquitination and proteasomal degradation by acting as a potential E3 ubiquitin ligase. IE1 physically interacts with Hes1 via amino acids (AA) 451–475, which are also essential for IE1-mediated Hes1 ubiquitination. In addition, Sp100A, an important component of PML nuclear bodies (PML-NBs), is identified as an additional ubiquitination substrate of IE1 ubiquitination. This study reveals a novel potential mechanism of HCMV induced neuropathogenesis and represents the first description of the potential E3 ubiquitin ligase activity of HCMV IE1.

## Results

### HCMV infection downregulates protein level of Hes1 in NPCs

**Fig 1:**
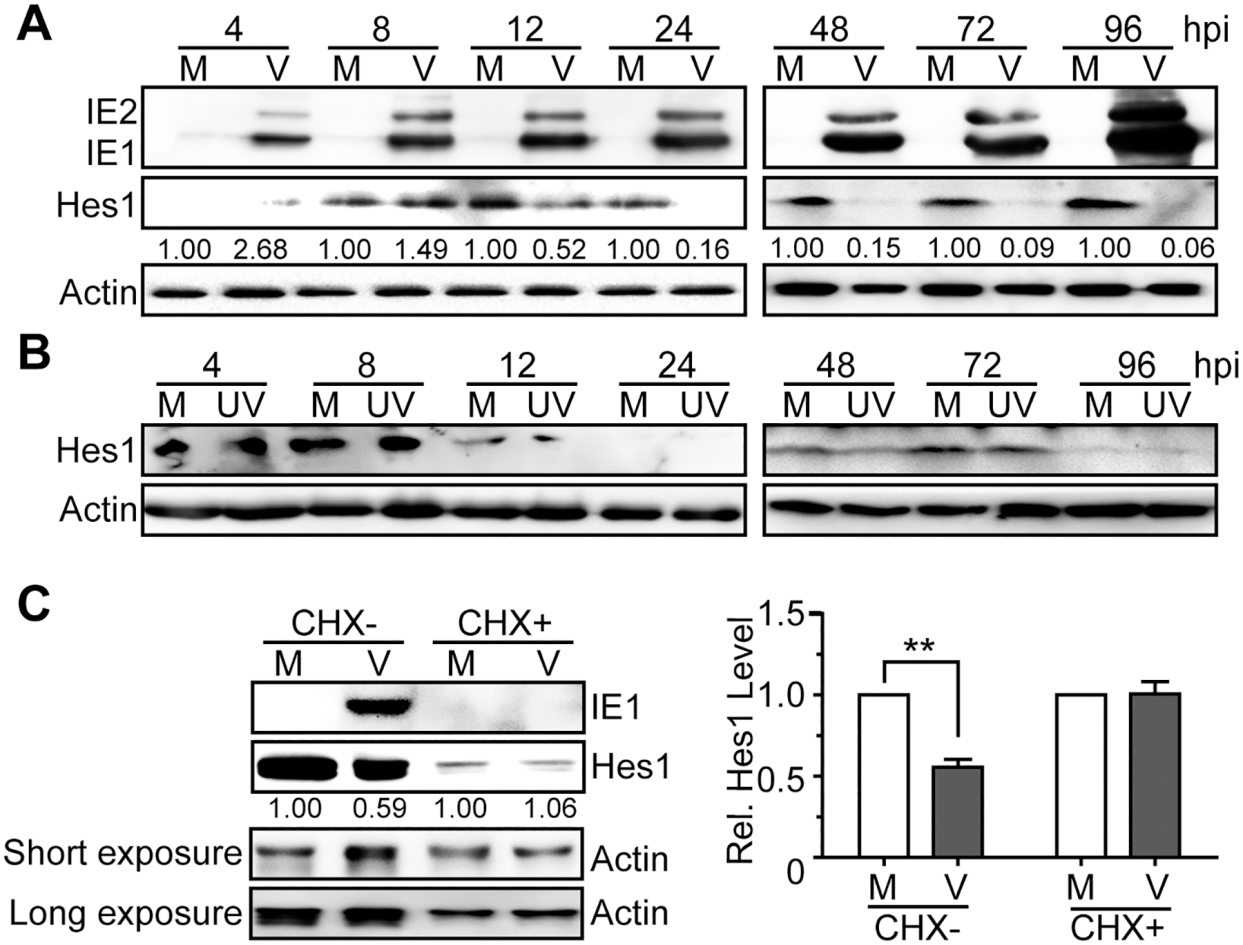
Downregulation of Hes1 protein during HCMV infection in NPCs requires *de novo* viral protein synthesis. (A-B) Hes1 protein level in NPCs during HCMV infection. NPCs were mock-infected (M) or infected with HCMV (V) or UV-inactivated HCMV (UV) at an MOI of 3. Cells were harvested at the indicated times post infection, and subjected to immunoblotting (IB). The values listed below the blots indicate the relative Hes1 protein levels compared to corresponding mock controls following β-actin normalization. (C) Effect of HCMV infection on Hes1 protein levels following CHX treatment. NPCs nucleofected with 0.25μg pCDH-Hes1 were pre-treated with 0.1mg/ml CHX (CHX+) or equal volume of solvent control DMSO (CHX-) for 1h, followed by mock (M)- or HCMV(V)-infection (MOI = 5) in the presence of CHX or DMSO. Cells were collected at 12hpi and subjected to IB. The values listed below the blots indicate the relative Hes1 protein levels compared to corresponding mock controls following β-actin normalization. Representative images from 3 independent experiments are shown (left), and the relative levels of Hes1 are presented as the mean ± SD (right). **, P≤0.01.

Human NPCs are fully permissive for HCMV replication [6–9], and our previous work has demonstrated that HCMV infection dysregulates NICD1 and Jag1 in NPCs, which are two essential components upstream of Hes1 in the Notch signaling pathway [5]. To investigate the effect of HCMV infection on Hes1, the HCMV Towne strain was used to infect NPCs at a multiplicity of infection (MOI) of 3. Protein levels of Hes1 were then examined by immunoblotting (IB) from 4hpi to 96hpi. In comparison to mock-infected cells, protein levels of Hes1 in infected NPCs were clearly decreased by 12hpi and were nearly undetectable from 24hpi to 96hpi (Fig 1A). UV-inactivated HCMV had no effect on Hes1 protein levels (Fig 1B), suggesting that the downregulation of Hes1 may depend on viral transcription and/or *de novo* synthesized viral gene products rather than input viral components. We have previously shown that Jag1 and NICD1 protein levels did not decrease before 16-24hpi (S1 Fig) [5], which was after Hes1 downregulation started (12hpi), indicating the Hes1 downregulation is not a consequence of Jag1 and NICD1 dysregulation in the IE phase of infection.

To confirm that Hes1 downregulation requires newly synthesized viral proteins, HCMV-infected NPCs were treated with the protein synthesis inhibitor cycloheximide (CHX). Due to the low levels of endogenous Hes1 in NPCs, 0.25μg of Hes1 expressing construct (pCDH-Hes1) was nucleofected into NPCs to enable Hes1 protein detection in the presence of CHX. As expected, the representative *de novo* synthesized viral protein IE1 was present in untreated HCMV-infected NPCs, but undetectable in CHX-treated cells. Similar to the data shown in Fig 1A, the Hes1 protein levels substantially decreased upon HCMV infection in the absence of CHX, but remained at similar levels between HCMV- and mock-infected NPCs upon CHX treatment (Fig 1C). Thus, *de novo* synthesized viral proteins, but not the input virus components, are necessary for Hes1 downregulation during HCMV infection.

Taken together, these data demonstrate that HCMV infection of NPCs results in depletion of the Hes1 protein via a mechanism that requires *de novo* viral protein synthesis.

### HCMV IE1 is sufficient and necessary for Hes1 downregulation

**Fig 2:**
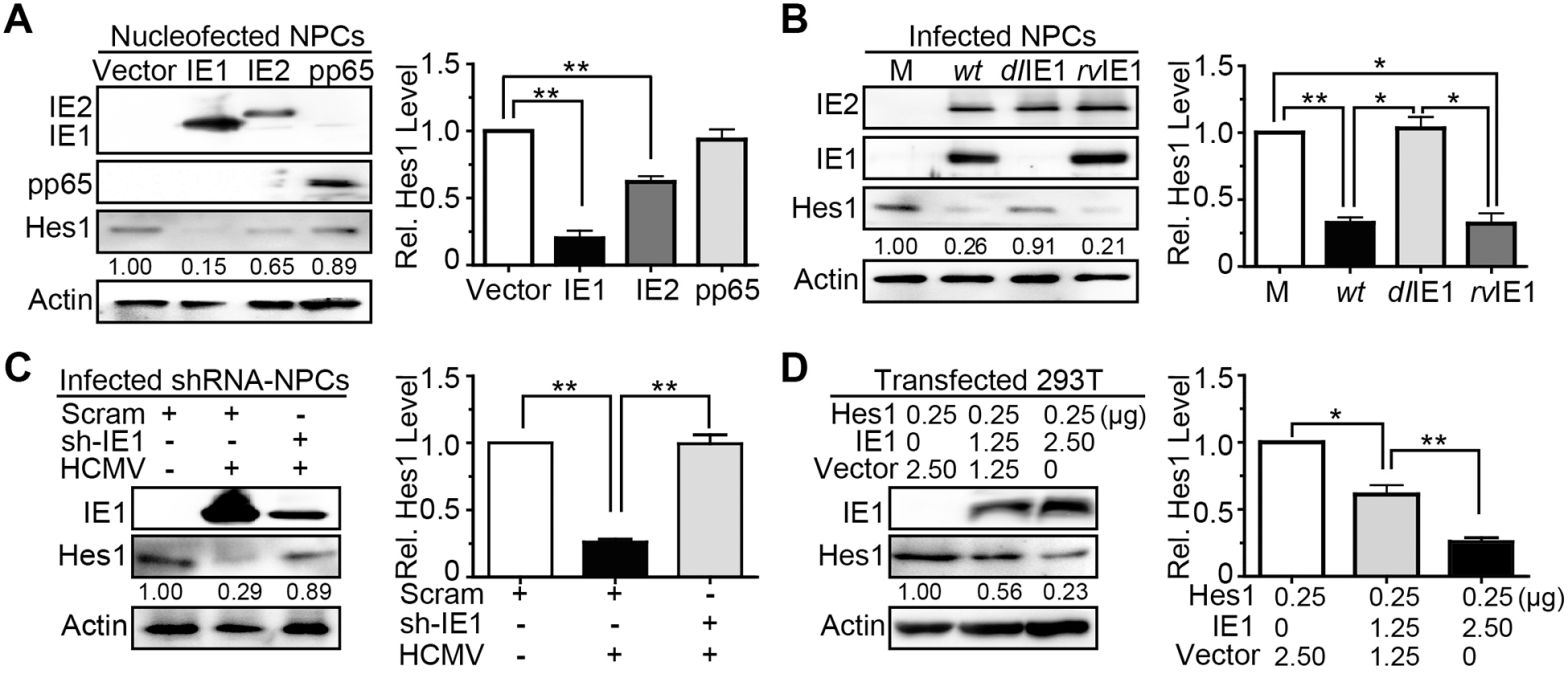
HCMV IE1 is sufficient and necessary to reduce Hes1 protein levels. (A) Effect of IE1, IE2 and pp65 expression on endogenous Hes1 protein levels in NPCs. At 48h following nucleofection with 5μg pCDH-GFP (vector), pCDH-IE1 (IE1), pCDH-IE2 (IE2) or pCDH-pp65 (pp65), NPCs were collected and subjected to IB for Hes1. (B) Effect of IE1 deletion on Hes1 protein downregulation during infection in NPCs. NPCs were mock-infected (M) or infected with TN*wt* (*wt*), TN*dl*IE1 (*dl*IE1) or TN*rv*IE1 (*rv*IE1) at an MOI of 10. Cells collected at 12hpi were subjected to IB. (C) Effect of IE1 knock-down on protein level of endogenous Hes1 during HCMV infection. At 48 h following transduction with lentiviruses expressing a scrambled shRNA (Scram) or an shRNA targeting IE1 (sh-IE1), NPCs were infected with HCMV (MOI = 1) for 24h and subjected to IB for Hes1 and IE1. (D) Effect of IE1 expression on protein levels of exogenous Hes1. At 48h following co-transfection with the indicated amount of pCDH-Hes1 (Hes1) and pEYFP-GFP (vector) or pEYFP-IE1 (IE1), 293T cells were collected and subjected to IB for Hes1. The values listed below the blots indicate the relative Hes1 protein levels compared to the corresponding controls following β-actin normalization. Representative images from 3 independent experiments are shown (left), and the relative levels of Hes1 are presented as the mean ± SD (right). *, P≤0.05; **, P≤0.01.

The fact that Hes1 downregulation occurs as early as 12hpi and the requirement for *de novo* viral protein synthesis suggest the potential involvement of HCMV proteins present during the IE phase of infection. Therefore, candidate viral gene products, including the major IE proteins (IE1 and IE2) and the most abundant input virus component pp65, were introduced into NPCs via nucleofection with plasmids pCDH-IE1, pCDH-IE2 and pCDH-pp65, respectively. Expression of pp65 displayed no significant effect on Hes1 protein level, consistent with the notion that the input components of HCMV virions are not involved in Hes1 downregulation induced by virus infection. In contrast, expression of IE1 or IE2 lead to markedly lower Hes1 protein levels (Fig 2A).

Considering that IE1 showed a stronger down-regulating effect on Hes1 than IE2, we focused our subsequent work on IE1. The effect of IE1 on protein level of Hes1 in the context of HCMV infection was further tested by comparing a recombinant IE1-deficient virus (TN*dl*IE1) to the parental wild-type (TN*wt*) and a “revertant” virus (TN*rv*IE1), respectively [38, 49, 50]. To overcome the replication defect of TN*dl*IE1 exhibited at low MOIs, the infection was performed at an MOI of 10, and the protein levels of Hes1 and IE1/2 were analyzed at 12hpi. As expected, IE1 was only present following the infection by TN*wt* and TN*rv*IE1 but not TN*dl*IE1, while IE2 levels were similar in all three infections. In TN*wt*- and TN*rv*IE1-infected NPCs, protein levels of Hes1 were downregulated to 32.5±0.04% and 32.0±0.08% of that in mock-infection (Fig 2B, right panel), respectively. In contrast, no obvious difference in protein levels of Hes1 between TN*dl*IE1- and mock-infected cells was observed (Fig 2B). Although IE2 expressed by itself seemed to also downregulate the protein level of Hes1 as shown in Fig 2A, it displayed virtually no capacity to alter Hes1 protein level in TN*dl*IE1 infected NPCs, perhaps due to its low abundance during infection and the relatively weak downregulating effect on Hes1 protein.

Consistent with our findings using the TN*dl*IE1 virus, shRNA-mediated IE1 knock-down in HCMV-infected NPCs restored the diminished Hes1 protein amount to normal level (Fig 2C). To exclude potential interference of endogenous Hes1 at the transcription level, a different cellular environment was applied to examine the downregulating effect of IE1 on exogenous Hes1. 293T cells were co-transfected with the constructs expressing IE1 (pEYFP-IE1) and Hes1 (pCDH-Hes1), and the protein levels were analyzed at 48 h post transfection (hpt). As shown in Fig 2D, the expressed IE1 reduced the exogenous Hes1 protein amount in a dose-dependent manner.

Taken together, IE1, either produced during HCMV infection or expressed in the absence of virus, downregulates the protein level of Hes1, and IE1 knock-down restores Hes1 protein level during virus infection. Notably, the protein level of Hes1 was minimally affected by IE2 in the absence of IE1 during the IE phase of infection. These results support that IE1 is sufficient and necessary for downregulation of the Hes1 protein during HCMV infection.

### IE1 directly interacts with Hes1 requiring AA451-475 of IE1

**Fig 3:**
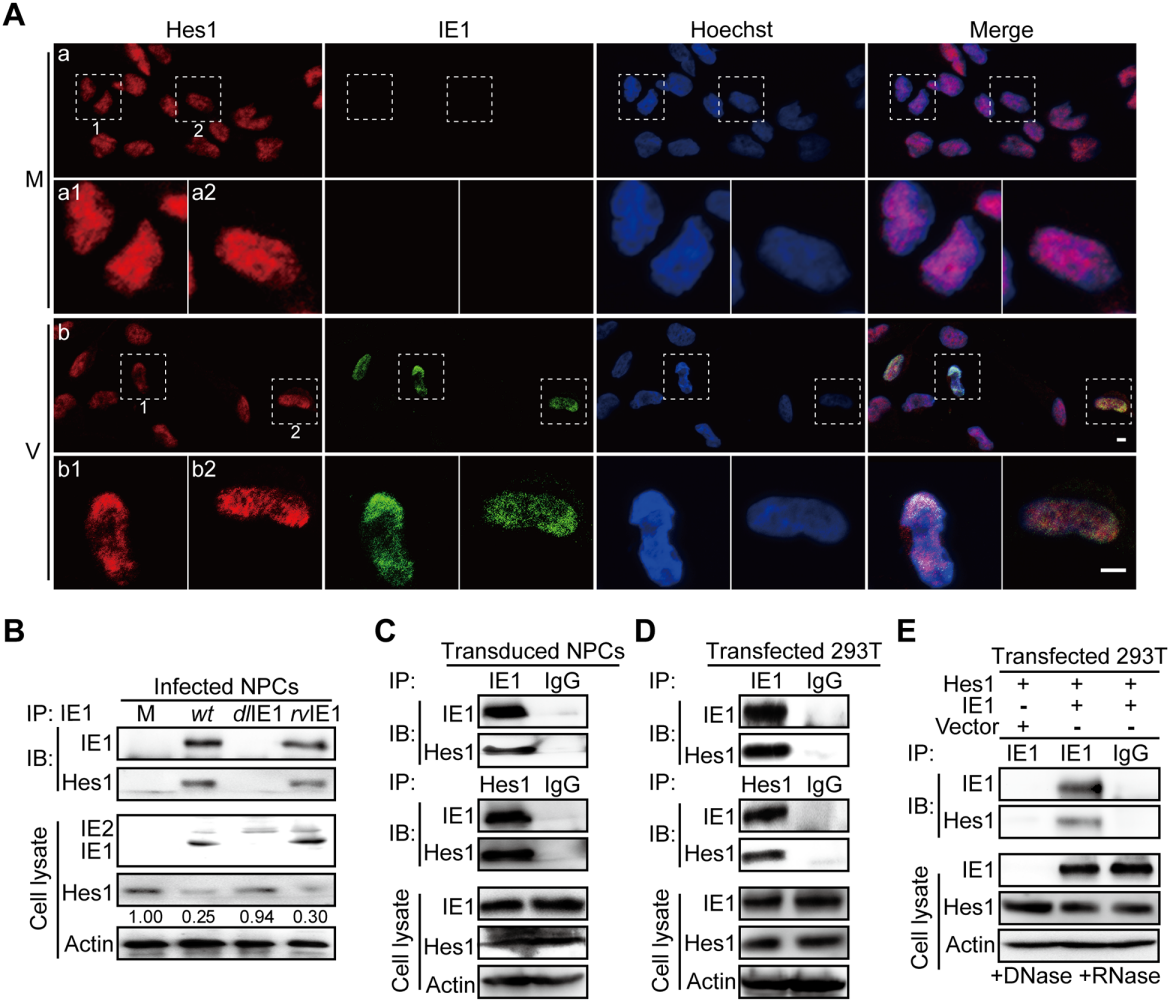
IE1 interacts with Hes1. (A) Subcellular localization of IE1 and Hes1 in NPCs. Following mock (M) or HCMV infection (V) at an MOI of 0.5, NPCs grown on coverslips were collected at 4hpi for immunofluorescence assay. Shown are representative images from 3 independent experiments. The boxed regions are presented with higher magnification. Scale bar, 5μm. (B) Interaction of IE1 and Hes1 in HCMV infected NPCs. NPCs were harvested at 12hpi from mock (M), TN*wt* (*wt*), TN*dl*IE1 (*dl*IE1) or TN*rv*IE1 (*rv*IE1) infection (MOI = 10), and subjected to IE1-directed IP analysis and subsequent IB for Hes1 and IE1. The indicated proteins were also examined in cell lysates. The values listed below the blots indicate the relative protein level of Hes1 compared to the corresponding mock control following β-actin normalization. Representative results from 3 independent experiments are shown. (C) Interaction of IE1 and Hes1 in transduced NPCs. NPCs were transduced with lentivirus expressing IE1. Cells were harvested at 72 hour post transduction and subjected to either IE1- or Hes1-directed IP analysis and subsequent IB for Hes1 and IE1, with normal IgG as a nonspecific antibody control. Representative results from 3 independent experiments are shown. (D) IE1/Hes1 physical interaction in transfected 293T cells. 293T cells co-transfected with 6μg pCDH-Hes1 and 6μg pEYFP-IE1 were collected 48hpt. Cells were analyzed by IE1- or Hes1-directed IP (normal IgG served as a nonspecific antibody control) and subsequent IB for Hes1 and IE1, respectively. The total IE1 and Hes1 protein levels in cell lysates were also assessed. (E) IE1/Hes1 physical interaction in the absence of DNA and RNA. Following co-transfection with 6μg pCDH-Hes1 (Hes1) and 6μg pEYFP-GFP (vector) or pEYFP-IE1 (IE1) for 48h, 293T cells were harvested. Cell lysates were treated with DNase and RNase prior to IE1-directed IP (normal IgG served as a nonspecific antibody control) and subsequent IB for IE1 and Hes1. The total IE1 and Hes1 protein levels in cell lysates were also assessed.

Having shown that IE1 expression was associated with downregulation of Hes1, we next investigated the underlying mechanism. Based on high resolution images obtained by two-photon microscopy, the Hes1 protein was distributed evenly across mock-infected NPC nuclei. However, upon HCMV infection, Hes1 was observed to relocate and concentrate at sub-nuclear areas where IE1 was present at 4hpi (Fig 3A).

The co-localization between IE1 and Hes1 suggests that these two proteins could be physically associated. To investigate a potential interaction between IE1 and Hes1 (IE1/Hes1 interaction), we carried out an immunoprecipitation assay (IP) in NPCs infected with TN*wt*, TN*dl*IE1 or TN*rv*IE1 for 12h. An IE1/Hes1 interaction along with Hes1 downregulation was only observed in TN*wt*- and TN*rv*IE1-, but not in TN*dl*IE1-infected cells (Fig 3B). To exclude the possibility that other viral proteins are mediating the IE1/Hes1 interaction, IP analysis was performed in the context of transduced NPCs expressing IE1 (Fig 3C) and transfected 293T cells co-expressing the two proteins (Fig 3D). Co-precipitated IE1 and Hes1 were detected by IB following IPs using Hes1- or IE1-specific antibodies, respectively. A normal IgG control was used for IP to rule out the possibility of non-specific binding (Fig 3C and 3D). Both IE1 and Hes1 are transcription factors: IE1 activates many different promoters and binds nucleosomes [51]; Hes1 binds its own promoter for auto-suppression [52]. To exclude the possibility that the IE1/Hes1 interaction is mediated via viral or cellular nucleic acids, cell lysates of transfected 293T cells were treated with DNase and RNase prior to IP analysis. As shown in Fig 3E, removal of DNA/RNA by DNase/RNase treatment (S2 Fig) did not alter the IE1/Hes1 interaction. This result indicates that IE1/Hes1 interaction is independent of nucleic acids and likely resulted from a direct protein-protein interaction.

**Fig 4:**
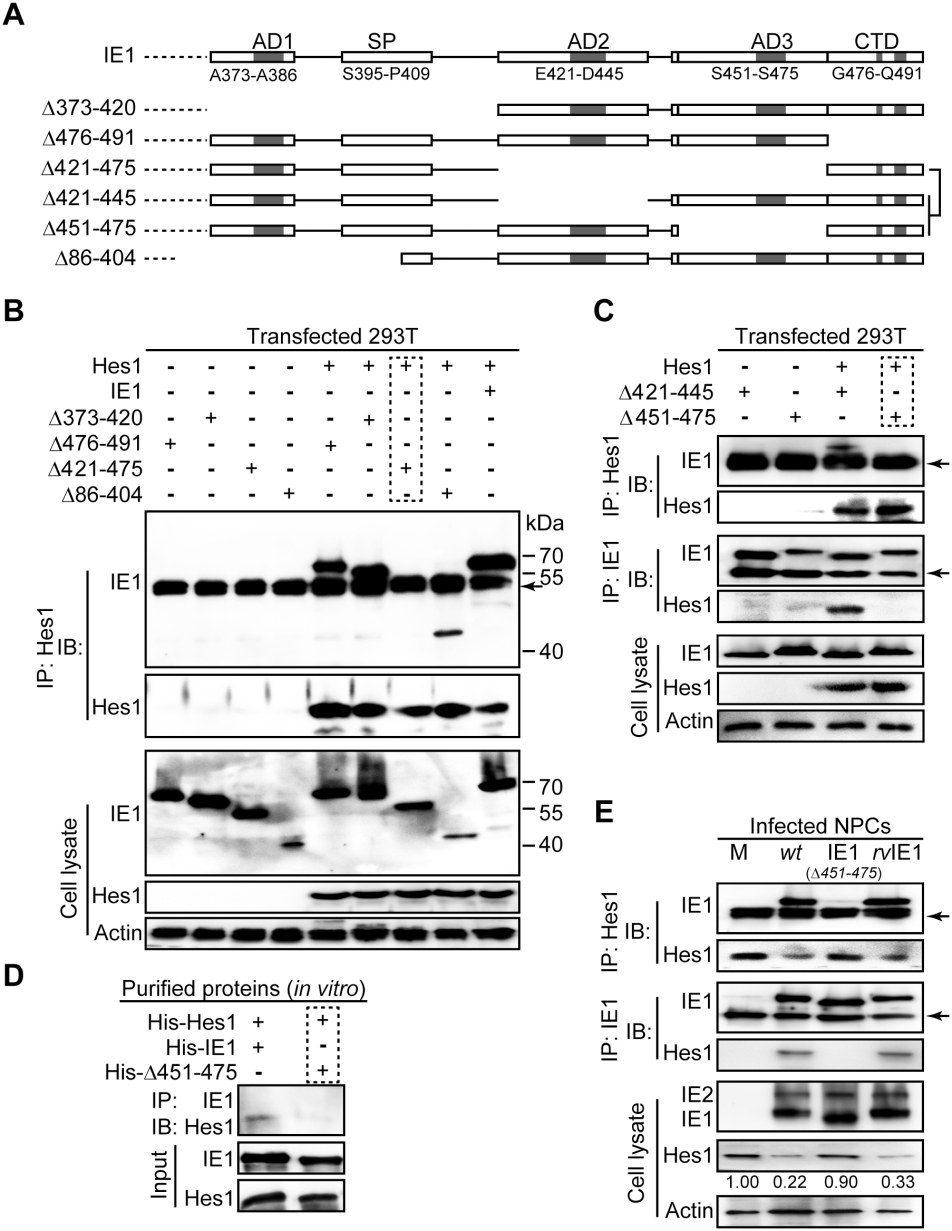
The IE1/Hes1 interaction requires AA451-475 of IE1. (A) Schematic diagram of wild-type IE1 and IE1 mutants used in this study. (B-C) IE1 region required for IE1/Hes1 interaction *in vivo*. Following co-transfection with 6μg plasmid pCDH-Hes1 and 6μg pEYFP -based plasmids expressing wild-type or mutant IE1 (Δ373–420, Δ476–491, Δ421–475, Δ86–404, Δ421–445 and Δ451–475) for 48h, 293T cells were sequentially processed for Hes1-directed IP and IB for IE1 and Hes1. The total IE1 and Hes1 protein levels in cell lysates were also assessed. Band corresponding to IgG heavy chains are indicated by arrows. (D) Interaction of purified IE1 or Δ451–475 and Hes1 *in vitro*. 1μg of each purified protein was mixed and subjected to pull down assay as described in Materials and Methods. (E) Interaction between IE1 or Δ451–475 and Hes1 in infected NPCs. Following mock (M), TN*wt* (*wt*), TN-IE1_(Δ451–475)_ (IE1_(*Δ451–475*)_) or TN*rvIE1* (*rvIE1*) infection at an MOI of 10, NPCs were harvested at 12hpi and cell lysates were subjected to either Hes1- or IE1-directed IP analysis and subsequent IB for IE1 or Hes1, respectively. The total IE1/2 and Hes1 protein levels in cell lysates were also assessed. The band corresponding to IgG heavy chain is indicated by an arrow.

The HCMV (Towne) IE1 protein comprises a total of 491 amino acids and can be roughly divided into an N-terminal (AA1-85, shared with IE2), a central/core (AA86-372) and a C-terminal (AA373-491) domain. Many interactions of IE1 and other proteins have been mapped to a region proximal to the C-terminus, which contains four short low complexity motifs including three acidic “domains” (AD1, AD2 and AD3) enriched in aspartic and glutamic acid, and a stretch of amino acids enriched in serine and proline (SP) [49]. The AD1 and SP motifs have been implicated in IE1/STAT2 binding [49, 53]. The terminal 16 amino acids following these four motifs are responsible for nucleosome targeting, and are thus named the chromatin tethering domain (CTD) [51] (Fig 4A).

To examine whether the IE1/Hes1 interaction depends on residues in the C-terminal domain of IE1, we tested a number of IE1 deletion mutants (Fig 4A). To this end, 293T cells were co-transfected with constructs expressing Hes1 and wild-type (IE1) or mutant IE1. The IE1/Hes1 interaction was observed at ratios of pCDH-Hes1: pEYFP-IE1 (Hes1:IE1) plasmid DNA of 1:1 and 1:5 (S3A Fig). A clearer result was obtained at the ratio of 1:1, and therefore a 1:1 ratio was used for further interaction analysis. The steady-state levels of all tested IE1 mutants were comparable to that of wild-type IE1 in cell lysates (Fig 4B and 4C). Subsequently, the interaction of IE1 mutants with Hes1 was investigated by IP analysis. To rule out any nonspecific binding, the plasmid pEYFP and normal IgG were used as the vector control and the nonspecific IP antibody control, respectively (S3B Fig). Due to the lack of commercial antibodies from different species for Hes1 and IE1, both the IP and the subsequent IB were performed with mouse monoclonal antibodies. This resulted in the detection of IgG heavy chains (Fig 4B, 4C and 4E, indicated by arrows). IE1 mutants Δ373–420, Δ476–491 and Δ86–404 were found to be co-precipitated with Hes1, but not Δ421–475 (Fig 4B). To exclude the potential interference of heavy chain bands, which were similar to the size of Δ421–475, and narrow down the interaction site of IE1, AA421-475 was further subdivided along the boundary of AD2 and AD3 into AA421-445 and AA451-475. One of the resulting IE1 mutants (Δ421–445) was still capable of binding Hes1, while the other one (Δ451–475) failed to interact, as determined by IP analysis (Fig 4C). Both mutants (Δ421–445 and Δ451–475) were clearly distinguishable from the heavy chain. To confirm that AA451-475 of IE1 is required for binding to Hes1 and to test whether the interaction occurs in the absence of other viral or host factors, His-tagged versions of Hes1, IE1, and IE1Δ451–475 were expressed in *E*. *coli*, purified using metal affinity chromatography, and used in an *in vitro* pull-down assay. As shown in Fig 4D, His-IE1 but not His-Δ451–475 pulled down His-Hes1. Furthermore, the IE1/Hes1 interaction, along with Hes1 downregulation, was not observed in NPCs infected with AA451-475 deleted virus (TN-IE1_*(Δ451–475)*_) (Fig 4E).

Taken together, our results indicate that IE1-mediated Hes1 downregulation is linked to direct interaction between the two proteins, which requires the AA451-475 region (comprising the AD3 motif) within the C-terminal domain of IE1.

### IE1 prompts Hes1 ubiquitination *in vivo*

Recently published findings have revealed that the IE1 protein of Rhesus cytomegalovirus, a close homolog of HCMV IE1, contains a secondary protein structure similar to the coiled-coiled domain of TRIM-25 [41]. Many TRIM proteins have E3 ubiquitin ligase activity [42], and several IE proteins of herpesviruses other than HCMV also function as E3 ubiquitin ligases, some of which share functional similarities with HCMV IE1 [43, 44, 47, 54, 55]. These data lead us to investigate whether HCMV IE1 mediates Hes1 downregulation through prompting its ubiquitination and proteasomal degradation.

**Fig 5:**
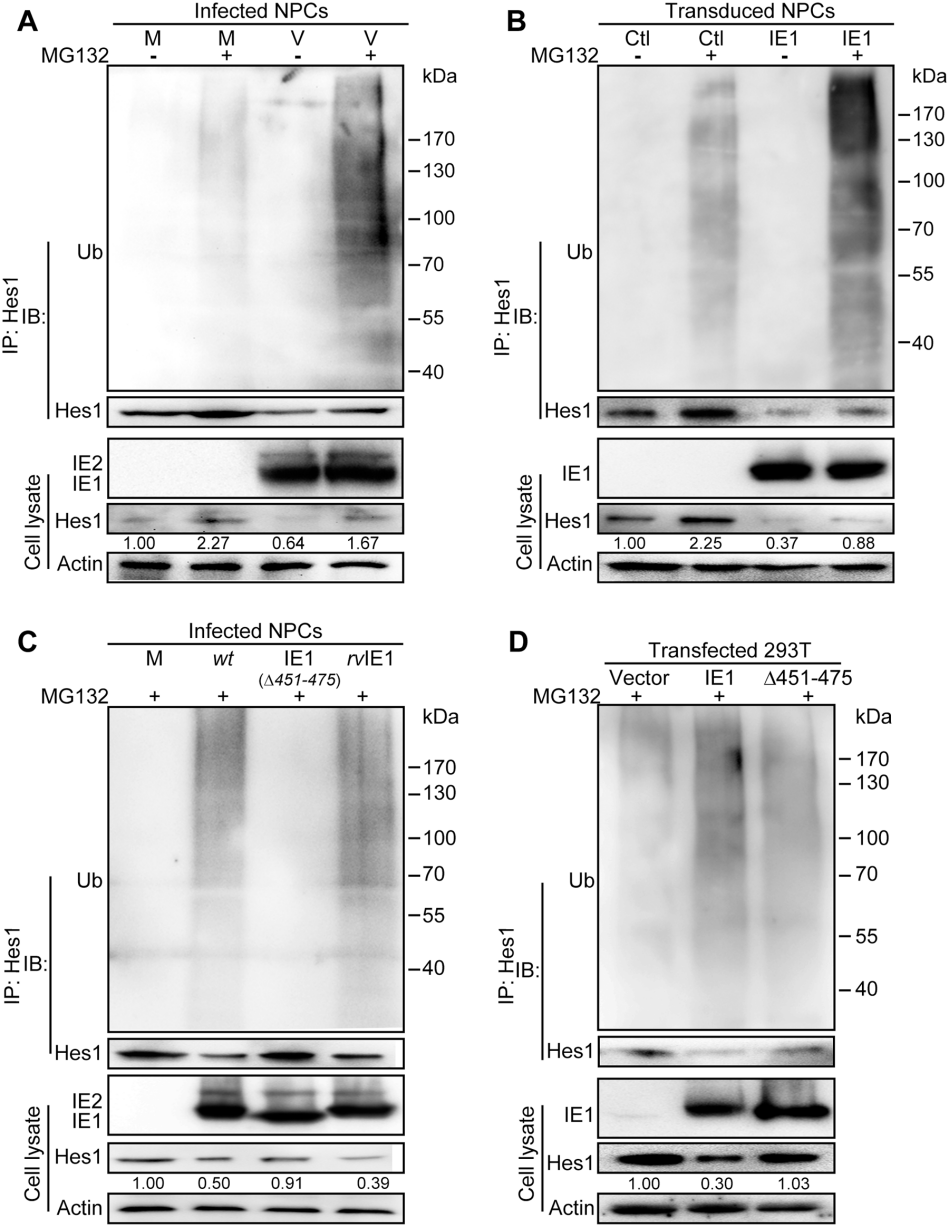
IE1 prompts Hes1 ubiquitination *in vivo*. (A) Effect of HCMV infection on ubiquitination of endogenous Hes1 in NPCs. Following mock (M) or HCMV infection (V) for 3h (MOI = 3), NPCs were treated with 12.5μM MG132 (+) or equivalent volume of DMSO (-) for 9h, and harvested at 12 hpi. Cell lysates were subjected to Hes1-directed IP and subsequent IB for ubiquitin (Ub) and Hes1. (B) Effect of IE1 on ubiquitination of endogenous Hes1 in transduced NPCs. NPCs were transduced with lentivirus expressing IE1 (IE1) or control (Ctl). Cells were treated with MG132 (+) or equivalent volume of DMSO (-) when GFP signal was clearly observed, harvested after being treated for 12 h, and subjected to Hes1-directed IP and subsequent IB for ubiquitin (Ub) and Hes1. (C) Effect of IE1 AA451-475 on ubiquitination of endogenous Hes1 in HCMV infected NPCs. NPCs were subjected to mock (M), TN*wt* (*wt*), TN-IE1_*(Δ451–475)*_ (IE1_*(Δ451–475)*_) or TN*rv*IE1 (*rv*IE1) infection at an MOI of 10. At 3hpi, NPCs were treated with 12.5μM MG132 (+) for 9h, and then harvested at 12 hpi. Cell lysates were subjected to Hes1-directed IP and subsequent IB for ubiquitin (Ub) and Hes1. (D) Effect of IE1 AA451-475 on ubiquitination of exogenous Hes1 in 293T cells. 293T cells were co-transfected with 0.25μg pCDH-Hes1 and 2.5μg pEYFP (vector), pEYFP-IE1 (IE1) or pEYFP-IE1_(Δ451–475)_ (Δ451–475). At 36 hpt, cells were treated with MG132 (+), and harvested after 12h treatment. Cell lysates were then subjected to Hes1-directed IP and subsequent IB for ubiquitin (Ub) and Hes1. For all the ubiquitination analysis *in vivo*, total levels of the indicated proteins were also examined in the corresponding cell lysates. The values listed below the Hes1 blots indicate the relative protein levels of Hes1 compared to the control(s) following β-actin normalization.

When levels of ubiquitinated Hes1 were examined in NPCs, Hes1 ubiquitination was observed in both mock- and HCMV-infected cells, indicating that the ubiquitin-proteasome pathway is involved in Hes1 protein regulation, which is concordant with previous observations in other cell types [16, 52]. Treatment with the proteasome inhibitor MG132 led to a substantial increase of Hes1 steady-state levels in cell lysates, as well as of ubiquitinated Hes1 in HCMV-infected NPCs, compared to DMSO controls and mock-infected NPCs (Fig 5A). Notably, MG132 treatment did not completely restore the Hes1 protein levels in the infected samples to levels found in mock control, indicating proteasomal degradation is likely not the only mechanism involved in Hes1 regulation. To examine the effect of IE1 on prompting Hes1 ubiquitination, NPCs were transduced with a lentivirus expressing IE1. After confirmation of IE1 expression, cells were treated with MG132 or DMSO, and then subjected to Hes1-directed IP followed by IB against ubiquitin. The levels of ubiquitinated Hes1 were increased in MG132-treated IE1-expressing NPCs compared to the MG132-treated IE1-negative cells (Fig 5B). Furthermore, the ubiquitination level of Hes1 protein in TN-IE1_*(Δ451–475)*_-infected NPCs was significantly lower than that in TN*wt*- and TN*rv*IE1-infected NPCs, while similar to that in mock-infected NPCs (Fig 5C). Consistently, IE1 but not Δ451–475 promoted exogenous Hes1 ubiquitination in transfected 293T cells (Fig 5D). These data suggested that IE1 downregulates Hes1 protein level by enhancing its ubiquitination and proteasomal degradation, and that these activities require AA451-475 of IE1.

Consistent with ubiquitination as a mechanism for Hes1 downregulation, expression of IE1 reduced the half-life of exogenous Hes1 from 19.1±2.9 min to 9.3±0.2 min in transfected 293T cells, while similar expression levels of IE1 mutant Δ451–475 did not alter Hes1 half-life (20.7±2.7 min) (S4 Fig).

### IE1 assembles Hes1 ubiquitination complex and mediates Hes1 ubiquitination *in vitro*

**Fig 6:**
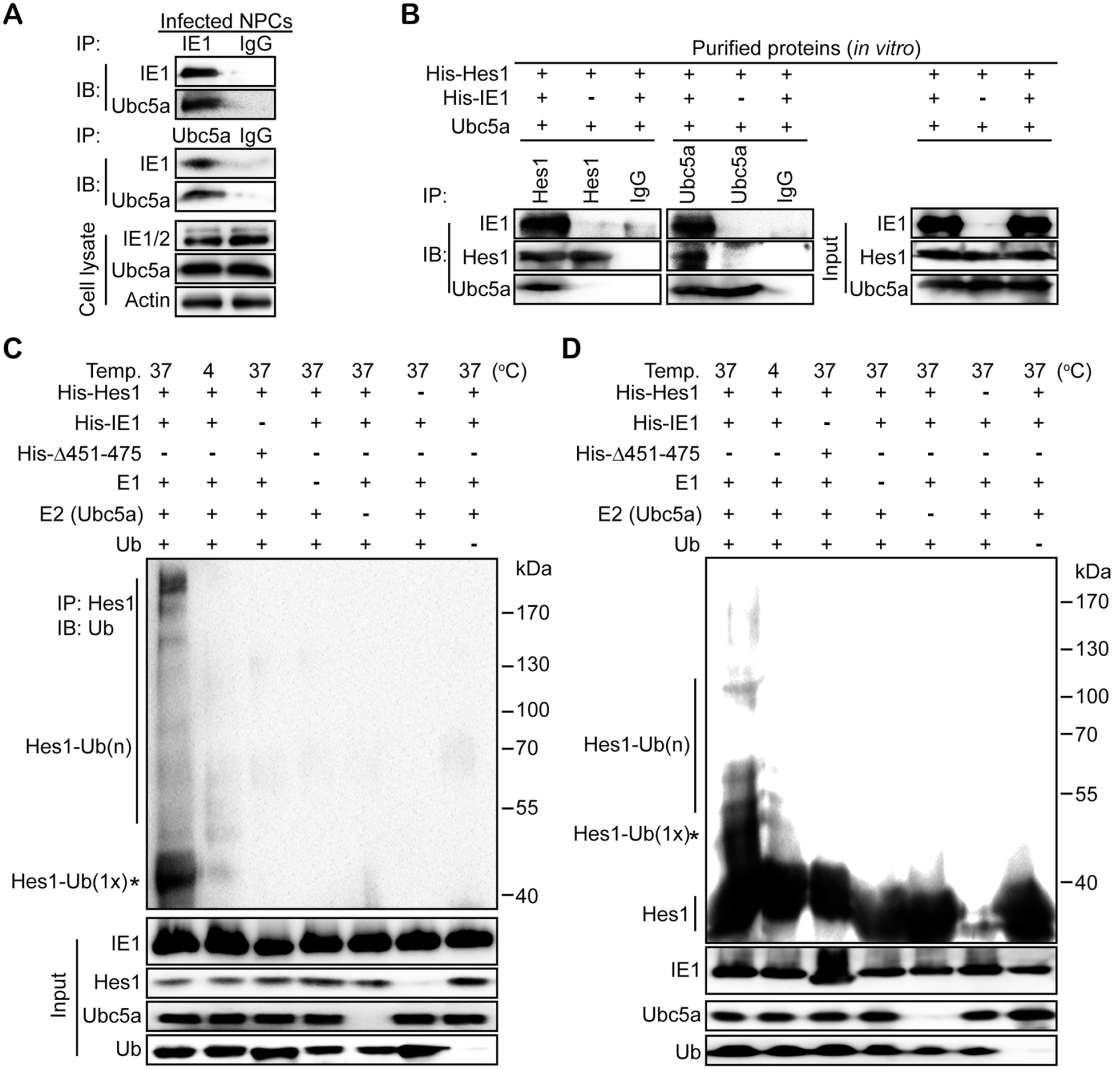
IE1 assembles a Hes1 ubiquitination complex and leads to ubiquitination of Hes1 *in vitro*. (A) Interaction between IE1 and Ubc5a in HCMV infected NPCs. NPCs were infected with HCMV at an MOI of 3, and harvested at 12hpi. Cell lysates were subjected to IE1- or Ubc5a-directed IP followed by IB against IE1 and Ubc5a. Normal IgG was applied as the nonspecific antibody control. (B) Effect of IE1 on assembling the Hes1 and Ubc5a ubiquitination complex. The purified His-Hes1 (1μg) was mixed with Ubc5a (1μg) in the presence or absence of purified His-IE1 (1μg). The mixtures were then subjected to Hes1- or Ubc5a-directed IP, followed by IB for the indicated component proteins. Normal IgG was applied as the nonspecific antibody control. The input components in the reaction were examined. (C-D) Effect of IE1 AA451-475 on ubiquitination of Hes1 *in vitro*. Purified His-tagged proteins (His-Hes1, His-IE1, His-Δ451–475) were mixed with ubiquitin-activating enzyme (E1), ubiquitin-conjugating enzyme Ubc5a (E2) and ubiquitin as described in Materials and Methods, and then incubated at the indicated temperature for 2 h to perform the *in vitro* ubiquitination reaction. The mixtures were subjected to Hes1-directed IP and subsequent IB for ubiquitin (Ub) (C). Alternatively, the products from *in vitro* ubiquitination reactions were directly subjected to IB with anti-Hes1 (D). According to protein size, mono-ubiquitinated Hes1 is indicated by an asterisk (*). The input components in the reaction were examined, and the indicated proteins are shown.

To ubiquitinate a substrate, it is necessary that an E3 ubiquitin ligase (E3) assembles the ubiquitination complex composed of E2 conjugating enzyme (E2), E3 and the substrate [56]. Therefore, we next investigated whether IE1 interacts with an E2 and Hes1 (substrate) and mediates the complex formation. Ubc5a serves as an E2 in HSV-1 ICP0 ubiquitinating Sp100A and KSHV RTA ubiquitinating IRF-7 and MyD88 [48, 55, 57], and an interaction between HCMV IE1 and Ubc5a was also observed in HCMV infected NPCs (Fig 6A). Thus, Ubc5a was used in the following assays as a potential E2 for IE1-mediated Hes1 ubiquitination. To exclude the possibility of influence from other viral or cellular components, the prokaryotically expressed proteins of His-Hes1, His-IE1 and His-Δ451–475 were purified. The purity of the purified recombinant proteins was examined by SDS-PAGE and Coomassie Brilliant Blue staining (S5A Fig). The purified proteins were incubated *in vitro* together with commercial Hes1- or Ubc5a-specific antibodies, and subjected to IP assay. The results showed that both Ubc5a (E2) and His-Hes1 (substrate) interact with IE1, and the His-Hes1/Ubc5a interaction was observed only in the presence of IE1, indicating that IE1 mediated the His-Hes1/Ubc5a interaction (Fig 6B). These data suggested that IE1 interacts with both Ubc5a and His-Hes1 to assemble a ubiquitination complex.

To confirm whether IE1 can mediate Hes1 ubiquitination as an E3 ligase, an *in vitro* ubiquitination reaction was also performed. The *in vitro* ubiquitination reaction included His-Hes1, His-IE1 or His-IE1Δ451–475 (4μg), ubiquitin activating enzyme (E1), Ubc5a, ubiquitin and the reaction buffer containing ATP. Following a 2 hours’ incubation at the indicated temperature, the reaction products were subjected to Hes1-directed IP followed by IB for ubiquitin. The addition of His-IE1 led to ubiquitinated His-Hes1, whereas ubiquitinated Hes1 was undetectable when the IE1 mutant His-Δ451–475 rather than the wild-type protein was present in the reaction. No Hes1 ubiquitination was observed in the absence of E1, Ubc5a or ubiquitin, which ruled out non-specific reactions (Fig 6C). To further confirm this result, we doubled the amount of the purified proteins (8μg) used in the *in vitro* ubiquitination assay and directly assayed the results by IB with anti-Hes1 antibody but without a prior IP. Ubiquitinated Hes1 was clearly detectable only in the presence of His-IE1 together with all other necessary components, but His-Δ451–475 failed to ubiquitinate His-Hes1 (Fig 6D). These data indicate that IE1 mediates Hes1 ubiquitination *in vitro* as a potential E3 ubiquitin ligase, and AA451-475 is required for IE1-mediated Hes1 ubiquitination.

Taken together, HCMV IE1 assembles Ubc5a and His-Hes1 to form a ubiquitination complex and mediates Hes1 ubiquitination as a potential E3 ubiquitin ligase. These processes require AA451-475 of the IE1 protein.

### IE1 prompts Sp100A ubiquitination and assembles a Sp100A ubiquitination complex

Sp100A is one of the major components of PML-NBs. Previously published data show that Sp100A is downregulated upon HCMV infection via proteasomal degradation, and IE1, which interacts with Sp100A, is potentially involved in this HCMV induced Sp100A downregulation [58, 59]. Sp100A is also a ubiquitination substrate of HSV-1 ICP0, an E3 ubiquitin ligase [55]. Moreover, both HCMV IE1 and HSV-1 ICP0 are IE proteins of herpesviruses and share certain similarities in their functions. Based on this evidence we next considered whether HCMV IE1 downregulates Sp100A via ubiquitination.

**Fig 7:**
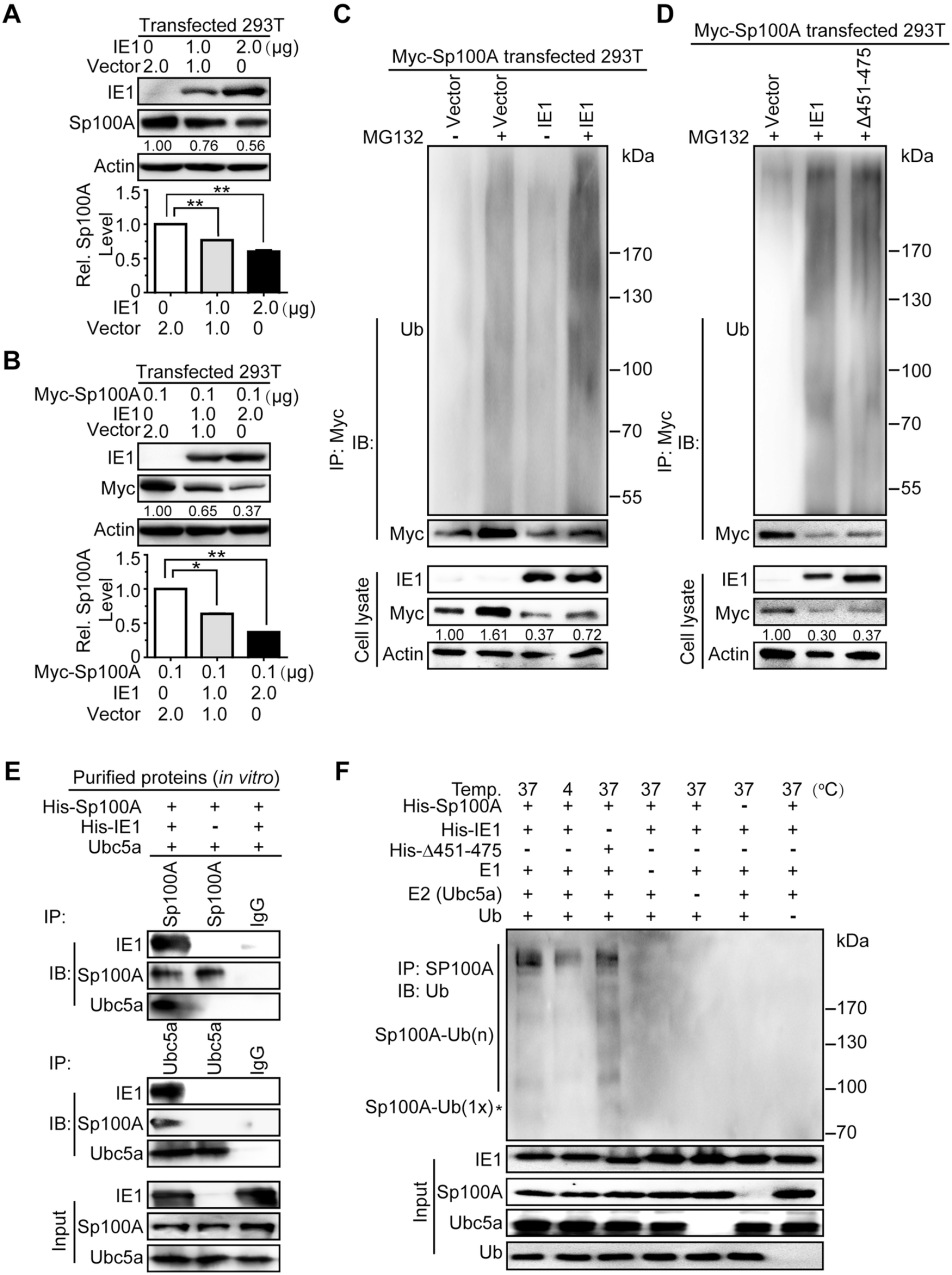
IE1 induces ubiquitination of Sp100A and assembles an Sp100A ubiquitination complex. (A-B) Effect of IE1 on the protein levels of endogenous and exogenous Sp100A in 293T cells. 293T cells were transfected with pEYFP-IE1 (IE1) or pEYFP (vector) in the absence (A) or presence (B) of pCMV-Myc-Sp100A (Myc-Sp100A). Cells were harvested at 48hpt for immunoblotting. Endogenous (A) or exogenous (B) Sp100A levels were examined by antibodies against Sp100A or Myc, respectively. The values listed below the blots indicate the relative protein levels of Sp100A compared to the controls following β-actin normalization. Representative images of IB from 3 independent experiments are shown (upper panel), and relative levels of Sp100A are presented as the mean ± SD (lower panel). **, P≤0.01. (C-D) Effect of IE1 on ubiquitination of exogenous Sp100A in 293T cells. 293T cells were transfected with 0.1μg pCMV-Myc-Sp100A (Myc-Sp100A) and 2μg pEYFP-IE1 (IE1), pEYFP-IE1_(Δ451–475)_ (Δ451–475), or pEYFP (vector). At 36hpt, cells were treated with MG132 (+) or DMSO (-) for 12 h. Cell lysates were subjected to Myc-directed IP and subsequent IB for ubiquitin (Ub) and Myc. The indicated proteins were also examined in the cell lysates. (E) Effect of IE1 on assembling the Sp100A ubiquitination complex. Purified His-Sp100A was mixed with Ubc5a in the presence or absence of purified His-IE1. The mixtures were then subjected to Sp100A- or Ubc5a-directed IP, followed by IB for the indicated proteins. Representative results from 3 independent experiments are shown. Normal IgG was used as the nonspecific antibody control. The input components in the reaction were examined, and the indicated proteins are shown. (F) Effect of IE1 on ubiquitination of Sp100A *in vitro*. Purified His-IE1 or His-Δ451–475 were mixed together with His-Sp100A, E1, E2 (Ubc5a) and ubiquitin as described above and in Materials and Methods, and incubated at the indicated temperature for 2h to perform *in vitro* ubiquitination reaction. The mixtures were subjected to Sp100A-directed IP and subsequent IB for ubiquitin (Ub). The input components in the reaction were examined, and the indicated proteins are shown. The mono-ubiquitinated Sp100A is indicated by an asterisk (*) according to the protein size.

Both the endogenous and exogenous Sp100A were downregulated by IE1 in a dose-dependent manner in 293T cells (Fig 7A and 7B), which is supported by previous observations [58, 59]. Since downregulation of exogenous Sp100A was more efficient than endogenous Sp100A, the ubiquitination level of exogenous Sp100A was subsequently examined by *in vivo* ubiquitination assay in 293T cells transfected with pCMV-Myc-Sp100A and pEYFP-IE1 or vector control. Cells were treated with MG132 or DMSO, and then subjected to Myc-directed IP followed by IB against ubiquitin. Following treatment with MG132, the expressed IE1 increased the ubiquitination level of Myc-Sp100A compared to the vector controls, suggesting that IE1 prompts Sp100A ubiquitination *in vivo* (Fig 7C). Δ451–475 and wild-type IE1 showed a similar capacity to ubiquitinate Sp100A (Fig 7D), which was different from that observed with the ubiquitination of Hes1.

To exclude interference from other cellular components in the *in vivo* system, ubiquitination complex formation and ubiquitination capacity were examined *in vitro* using purified His-Sp100A and His-IE1 (S5B Fig). His-IE1 bound to both Ubc5a and His-Sp100A (substrate), and mediated the interaction between them (Fig 7E), indicating IE1 plays an essential role in formation of the Sp100A ubiquitination complex. Moreover, ubiquitinated Sp100A was detected in the presence of His-IE1, as well as His-Δ451–475, together with all other necessary components (Fig 7F). Importantly, deletion of AA451-475 abolished the ubiquitination capacity of IE1 on Hes1 (Figs 5C, 5D and 6C, 6D), but had no influence on IE1-induced Sp100A ubiquitination (Fig 7D and 7F).

**Fig 8:**
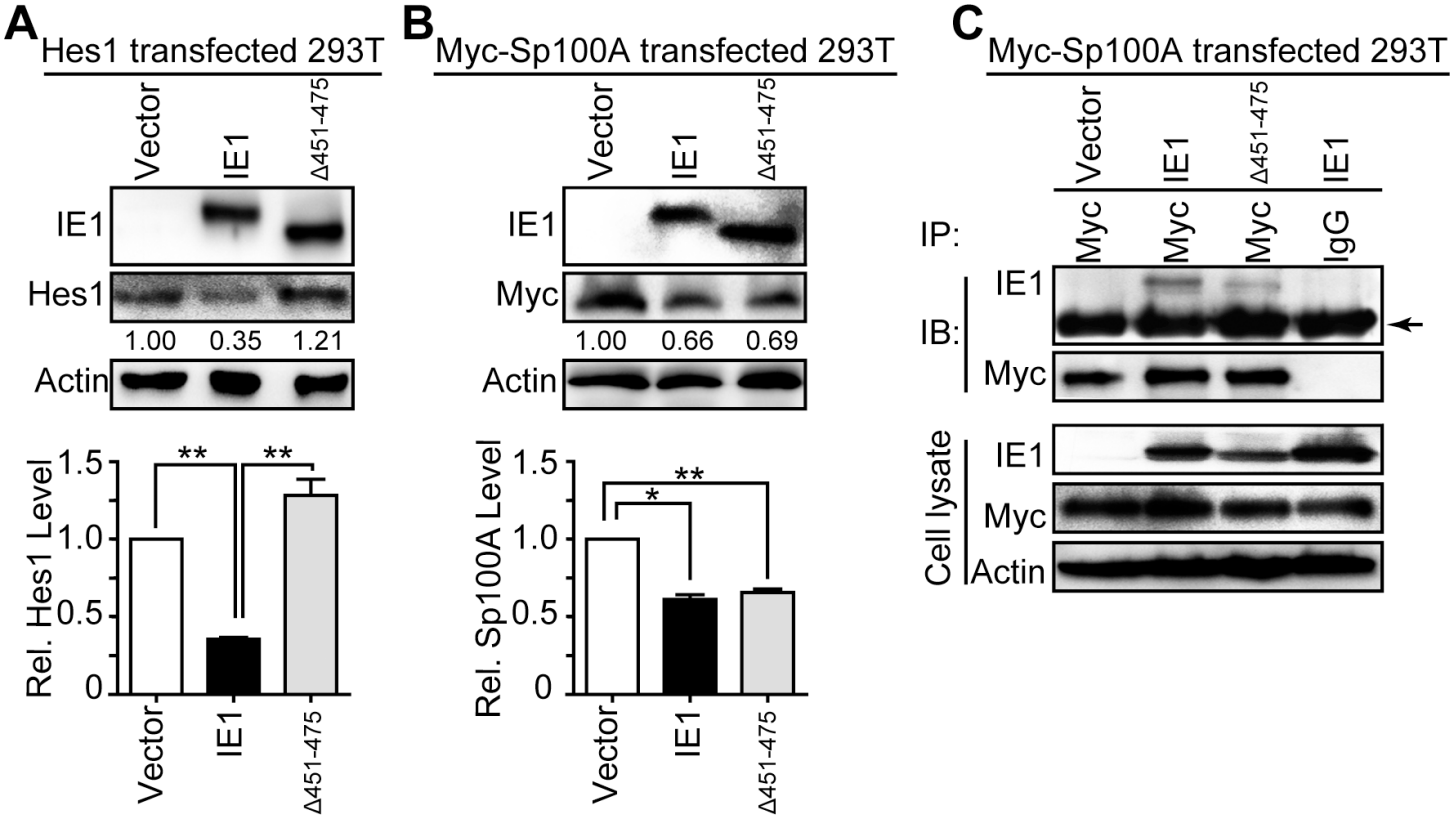
IE1 AA451-475 is dispensable for Sp100A interaction and downregulation. (A-B) Effect of Δ451–475 on protein levels of exogenous Hes1 and Sp100A in 293T cells. 293T cells were co-transfected with 0.25μg pCDH-Hes1 (Hes1) (A) or pCMV-Myc-Sp100A (Myc-Sp100A) (B) along with 2.5μg pEYFP (vector), pEYFP-IE1 (IE1) or pEYFP-IE1_(Δ451–475)_ (Δ451–475). Cells were harvested at 48hpt for IB to examine the protein levels of Hes1 (A) or Sp100A (B). The values listed below the blots indicate the relative protein levels of Hes1 or Sp100A compared to the controls following β-actin normalization. Representative images from 3 independent experiments are shown (upper panel), and relative protein levels are presented as the mean ± SD (lower panel). *, P≤0.05; **, P≤0.01. (C) Interaction of IE1 or Δ451–475 with Sp100A in 293T cells. 293T cells were transfected with 6μg pCMV-Myc-Sp100A (Myc-Sp100A) and 6μg pEYFP (vector), pEYFP-IE1 (IE1) or pEYFP-IE1_(Δ451–475)_ (Δ451–475). Cells were harvested at 48hpt and cell lysates were subjected to Myc-directed IP and subsequent IB for IE1 and Myc. Normal IgG was applied as nonspecific antibody control, and the indicated proteins were examined in the cell lysates. The band corresponding to IgG heavy chain is indicated by an arrow.

The differential effect of IE1 AA451-475 deletion on Hes1 and Sp100A prompted us to analyze whether AA451-475 is also required for downregulation of Sp100A by IE1. Consistent with the ubiquitination capacity, IE1 mutant Δ451–475 failed to reduce the protein levels of exogenous Hes1 in 293T cells (Fig 8A). However, Δ451–475 still downregulated exogenous Sp100A (Fig 8B), and accordingly, retained a capacity for binding to Sp100A (Fig 8C). These data indicate AA451-475 is not required for either Sp100A downregulation or the IE1/Sp100A interaction.

Taken together, consistent with regulation of Hes1 by IE1, IE1 initiates Sp100A ubiquitination and assembles an Sp100A ubiquitination complex. Moreover, while AA451-475 of IE1 are necessary for the IE1/Hes1 interaction, this region is dispensable for IE1-mediated Sp100A downregulation and IE1/Sp100A interaction.

## Discussion

As a critical downstream effector in Notch signaling, the Hes1 protein regulates the fate of NPCs by repressing the transcription of pro-neural genes and thus plays an essential role in maintaining the stem cell status of NPCs, governing their differentiation towards neurons or glia, and controlling fetal brain development [16, 17]. Congenital HCMV infection is one of the most common causes of neurological disabilities in children. However, the relationship between Hes1 regulation and virus infection, in particular whether and how HCMV regulates Hes1, remained unknown. In the present study, we report for the first time that HCMV infection downregulates Hes1 protein at the level in human NPCs through IE1 via a newly identified function, which may be a key mechanism that contributes to fetal brain maldevelopment caused by congenital HCMV infection.

Hes1 protein synthesis is highly regulated at the transcription level and by rapid degradation through the ubiquitin-proteasome pathway [52]. To exclude the possibility of potential interference of endogenous Hes1 transcription in NPCs, exogenous Hes1 was constitutively expressed in 293T cells using construct pCDH-Hes1. Under the control of the HCMV major IE promoter, the transcription level of Hes1 was elevated in the presence of HCMV IE1, but the protein level of Hes1 was still decreased, indicating the strong downregulating effect of IE1 on Hes1 at the post-translational level. In addition, IE1 physically interacts with Hes1, thus leading us to ask whether IE1 downregulates Hes1 through the ubiquitin-proteasome pathway.

The ubiquitin-proteasome pathway has been implicated in numerous physiological processes and disease outcomes, including regulation of genes controlling fetal brain development, and has been associated with neurodegenerative diseases [60, 61]. Additionally, the proteasome pathway is also one of the cellular machineries commonly hijacked by viruses to degrade cellular or viral proteins to favor virus replication [62–64]. For example, HSV-1 utilizes proteasome-mediated ubiquitination-independent proteolysis for successful target cell entry [65], and KSHV relies on ubiquitin-dependent proteasome degradation for viral entry and intracellular trafficking in endothelial cells [66]. The ubiquitination-proteasome pathway is also utilized by HCMV to promote viral gene expression [67].

Ubiquitination relies on three essential steps, each depending on particular enzyme(s): (1) activation of ubiquitin by ubiquitin-activating enzyme (E1); (2) transfer of the activated ubiquitin to ubiquitin-conjugating enzyme (E2) and formation of an E2-Ub thioester; and (3) formation of an isopeptide bond between ubiquitin and the substrate protein by ubiquitin protein ligase (E3) [68]. To accomplish step 3, E3 ubiquitin ligase assembles a ubiquitination complex by interacting with both the E2-Ub thioester and the specific substrate, and then mediates the transfer of ubiquitin from the E2-Ub thioester to the substrate [69]. This specific substrate recognition and interaction of E3 ubiquitin ligase also confers the specificity of ubiquitination [70].

Many viral proteins have been identified to possess E3 ubiquitin ligase activity, and several members of the herpesvirus also encode their own E3 ubiquitin ligases, such as ICP0 of HSV-1, ORF61P of VZV, K3/K5 and RTA of KSHV, as well as mK3 and ORF75c of murine gamma herpesvirus 68 (MHVγ68) [54, 71]. Although no HCMV-, or even β-herpesvirus-, encoded E3 ubiquitin ligase has been identified, the following data imply that HCMV IE1 might be an E3 ubiquitin ligase: (1) all identified E3 ubiquitin ligases of herpesviruses are IE proteins [54]; (2) HCMV IE1 involves in HCMV infection induced Sp100A downregulation through proteasomal pathway, and interacts with Sp100A, which is also a target of HSV-1 encoded E3 ubiquitin ligase ICP0 [55, 58, 59, 72]; (3) the central core domain of the Rhesus cytomegalovirus IE1, the HCMV IE1 homolog, shares certain secondary structure similarity with the TRIM proteins, many of which function as E3 ubiquitin ligases [41]; and (4) most viral E3 ubiquitin ligases belong to the RING E3 family, whose activity depends on the RING domain—two zinc atoms complexed with the cysteine/histidine residues in a ‘cross-brace’ manner [73]. Interestingly, IE1 contains a zinc finger motif (HX_2_HXFX_3_LX_2_CX_4_C, AA267-284) with two cysteines and two histidines (underlined) capable of binding Zn_2_^+^ [37]. Our results and these known features of HCMV IE1 structure lead us to speculate that this viral protein may function as an E3 ubiquitin ligase and downregulate Hes1 and Sp100A via ubiquitination.

IE1 does not contain any known canonical E3 ubiquitin ligase motifs, including HECT domain, RING finger and U-box [68]. However, proteins without these structures may also act as E3 ubiquitin ligases [73]. For example, RTA of KSHV functions as an E3 ubiquitin ligase regulating IRF7, K-RBP, MyD88, LANA-1 and KbZIP, but lacks any of the known E3 motifs [48, 57, 71]. The different families of E3 ubiquitin ligases regulate versatile E3 substrates via various E3 functional mechanisms, and their substrate recognition specificity makes E3 ubiquitin ligases share very few unified sequences or structure similarities. Thus, the most convincing and efficient method to identify a specific protein as an E3 ubiquitin ligase is through biochemical methods, including the *in vitro* ubiquitination assay [68].

Our studies have provided several findings to support the role of IE1 as a potential E3 ubiquitin ligase: (1) the purified IE1 ubiquitinates substrates Hes1 and Sp100A *in vitro* in the presence of E1, Ubc5a, ubiquitin and ATP, and the absence of any of these components resulted in a failure of IE1 to function in the ubiquitination assay; (2) IE1 interacts with Ubc5a and the substrates, which is one of the common features of E3 ubiquitin ligases [68, 73]; and (3) the ubiquitination reaction requires the formation of the E2/E3/substrate complex[56], and our data demonstrate that IE1 assembles Ubc5a and substrate (Hes1 and Sp100A) to form a tripartite complex. Ubc5a was used as the E2 in the *in vitro* ubiquitination reaction because it is recruited as an E2 enzyme when HSV-1 ICP0 or KSHV RTA ubiquitinate Sp100A or IRF-7 and MyD88, respectively [48, 55, 57]. Taken together, these results demonstrate that HCMV IE1 assembles the Ubc5a and Hes1 or Sp100A to form a ubiquitination complex, and mediates the ubiquitination of the substrates, thus functioning as a potential E3 ubiquitin ligase.

AD3 (AA 451–475) in the C-terminal domain of IE1 is essential for the interaction of IE1/Hes1 but not IE1/Sp100A. IE1 Δ451–475 failed to interact with and downregulate the Hes1 protein, but retained its effect on Sp100A. The E3 ubiquitin ligase requires the assembly of the ubiquitination complex by binding E2 and substrate, thus AA451-475 of IE1 is a Hes1 binding site rather than the active site of E3 ubiquitin ligase activity. According to the structure of IE1, AA280 and AA284 cysteines within the putative zinc finger motif may be the potential catalytic sites of E3 activity. Alternatively, the IE1 zinc finger possibly mediates the interaction of IE1 and Ubc5a, similar to cellular E3 RNF125 (also known as TRAC-1) [74]. However, the E3 catalytic center of IE1 may be located elsewhere and thus requires further investigation.

Multiple host cellular factors restrict viral replication. For instance, PML-NBs, consisting of PML, Daxx, Sp100A and other proteins, suppress HCMV replication as intrinsic anti-viral structures [75, 76]. Depletion of Sp100A, an important PML-NB component, enhances IE1/2 expression and HCMV replication [58]. Correspondingly, viruses target the intrinsic cellular defense proteins through viral encoded E3 ubiquitin ligases to counteract host restriction and favor viral replication [54, 55, 62]. HCMV has been reported to downregulate Sp100A via the proteasomal pathway, and IE1 has been implicated in this process [58, 59], which supports our finding that IE1 prompts Sp100A ubiquitination and proteasomal degradation as an E3 ligase.

Downregulation of Sp100A protein occurred in both Towne and AD169 infected fibroblasts, but at different timing. Towne infection started to decrease Sp100A protein as early as 12 hpi, but obvious Sp100A protein downregulation was observed till the late stage (72 hpi) of AD169 infection [58, 59]. IE1 proteins of Towne and AD169 differ at only AA68 and AA394, Arginine and Alanine for HCMV-IE1, and Histidine and Valine for AD169-IE1, respectively. Both variations employ amino acids with similar characters, and locate outside of the potential E3 catalytic regions. Therefore, IE1 of both strains are presumed to similarly boost Sp100A degradation as E3 to facilitate viral replication, which is partially supported by the above mentioned studies [58, 59]. On the contrary, host cells also upregulate Sp100A to activate the intrinsic anti-viral response upon viral infection, and different viruses possibly trigger various levels of cellular response, as well as Sp100A upregulation. Taken together, the variation of Sp100A downregulation timing observed upon Towne and AD169 infection is resulted by the different cellular and viral counteraction profiles induced by different viruses. But this hypothesis requires further confirmation.

Our preliminary data also showed that the expression levels of IE1/2 were decreased in Hes1 overexpressing human embryonic lung fibroblast cells (HELs) (S6 Fig). The downregulation of Hes1 through HCMV IE1 is a probable mechanism to counteract the suppression of Hes1. However, the mechanism by which Hes1 regulates HCMV replication remains unknown and requires further investigation.

In summary, (i) HCMV infection downregulates Hes1 protein through IE1; (ii) IE1 directly interacts with Hes1, prompting Hes1 ubiquitination and proteasomal degradation, and thus reduces the half-life and the steady-state level of the Hes1 protein; and (iii) Sp100A is identified to be another target of HCMV IE1-mediated ubiquitination. In addition, IE1 assembles E2 (Ubc5a) and substrate (Hes1 and Sp100A) to form a ubiquitination complex, and further mediates their ubiquitination. The regulation of Hes1 by IE1 requires AA451-475, which mediate binding to Hes1, but this region is not required for regulation of Sp100A. Our study not only suggests an important mechanism for fetal brain development disorders induced by congenital HCMV infection, but also reveals a novel unanticipated function of HCMV IE1 as a potential E3 ubiquitin ligase.

## Materials and methods

### Ethics statement

Human neural progenitor cells (NPCs) and human embryonic lung fibroblast cells (HELs) were isolated from postmortem fetal embryo tissues of brain and lung, respectively. The original source of the anonymized tissues was Zhongnan Hospital of Wuhan University (China). The cell isolation procedures and research plans were approved by the Institutional Review Board (IRB) (WIVH10201202) according to the Guidelines for Biomedical Research Involving Human Subjects at Wuhan Institute of Virology, Chinese Academy of Sciences. The need for written or oral consents was waived by IRB.

### Cells and cell culture

NPCs were isolated and maintained in the laboratory as described previously [10]. Briefly, hippocampus, bilateral ventricular and subventricular zone tissues were isolated from the brain, washed with Hank’s Balanced Salt Solution (HBSS) supplemented with antibiotics (1,000U/ml of penicillin, 1mg/ml streptomycin, Life Technologies), diced with scalpel blades and eye scissors, digested with DMEM-F12 media (Life Technologies) containing 2.5U/ml papain (Worthington), 40U/ml dispase II (StemCell Technologies) and 1U/ml DNase I (Qiagen) at 37°C for 20 min, mixed with 90% Percoll in phosphate-buffered saline (PBS), and centrifugated at 1,500g for 15 min. The NPCs were obtained from the top Percoll layer. The growth medium (GM) used for NPCs was DMEM-F12 supplemented with 2mM GlutaMax, penicillin-streptomycin (100U/ml and 100mg/ml) 50mg/ml gentamycin, 1.5mg/ml amphotericin B (all from Life Technologies), 10% BIT9500 (StemCell Technologies), 20ng/ml epidermal growth factor and 20ng/ml basic fibroblast growth factor (Prospec). For maintenance, half of the NPCs culture medium was replaced with fresh GM every two days, and the replaced medium was liberated from cell debris and used as conditioned medium (CM) [10].

For HELs isolation, lung tissues were prepared under sterile conditions, rinsed with PBS containing 1,000U/ml of penicillin and 1mg/ml streptomycin, diced into 1mm^3^ fragments, digested with 0.25% trypsin (Life Technologies) at 37°C for total 15 min with gentle shaking for three times, then mixed with equal volume of MEM media containing 10% fetal bovine serum (FBS, Life Technologies), and centrifuged at 500g for 10 min. The HELs were obtained from the pellet.

HELF-IE1, the cell line stably expressing HCMV IE1, was obtained by lentivirus (pCDH-puro-IE1) transduction and puromycin (8μg/ml, Sigma) selection on HELFs, which are immortalized human embryonic lung fibroblast cells (kindly provided by Dr. Jason J. Chen at Columbia University). Expression of IE1 was confirmed by immunoblotting and the resulting HELF-IE1 cell line was maintained in the presence of puromycin (4μg/ml). Human embryonic kidney (HEK) 293T cells were purchased from ATCC (CRL-11268). The cells were cultured in appropriate medium (MEM for HELs and HELF-IE1, and DMEM for 293T) supplemented with 10% FBS, penicillin-streptomycin (100U/ml and 100mg/ml) at 37°C in a humidified atmosphere containing 5% CO_2_.

### Plasmids

For exogenous protein expression in NPCs and 293T cells, pCDH-CMV-MCS-EF1-copGFP (referred to as pCDH, System Biosciences), pCMV-Myc and pEYFP-N1 (referred to as pEYFP, Clontech Laboratories) were used. The coding sequences of human Hes1, Sp100A, HCMV IE1 (UL123), IE2 (UL122) and pp65 (UL83) were cloned into the vectors to generate pCDH-Hes1, pCMV-Myc-Sp100A, pCDH-IE1, pCDH-IE2, and pCDH-pp65, respectively. pEYFP constructs expressing wild-type IE1 and IE1 mutants lacking specific segments of amino acids comprised pEYFP-IE1, pEYFP-IE1_(Δ86–404)_, pEYFP-IE1_(Δ373–420)_, pEYFP-IE1_(Δ421–445)_, pEYFP-IE1_(Δ421–475)_, pEYFP-IE1_(Δ451–475)_ and pEYFP-IE1_(Δ476–491)_. Hes1, Sp100A, wild-type IE1 and IE1_(Δ451–475)_ were also cloned into pET28a with a hexa-histidine- (His-) tag fused to their N-termini for prokaryotic expression and purification of the target proteins, including pET-Hes1 (His-Hes1), pET-Sp100A (His-Sp100A), pET-IE1 (His-IE1) and pET-IE1_(Δ451–475)_ (His-Δ451–475). For IE1 knock-down, lentiviruses expressing a short hairpin RNA (shRNA) targeting IE1 (sh-IE1) and a non-specific scrambled shRNA control (Scram) were applied. The sequences of shRNAs are as follows: sh-IE1, GCT GTG CTG CTA TGT CTT AGA CTC GAG TCT AAG ACA TAG CAG CAC AGC TTT TTG; Scram, CCT AAG GTT AAG TCG CCC TCG CTC GAG CGA GGG CGA CTT AAC CTT AGG TTT TTG.

### Viruses and infection

The viruses applied for infection comprised wild-type HCMV Towne strain (referred to as HCMV, ATCC VR-977), and its bacterial artificial chromosome (BAC)-derived recombinant viruses including TN*wt* (wild-type), TN*dl*IE1 (IE1 deleted), TN*rv*IE1 (IE1 revertant) and TN-IE1_(Δ451–475)_ (IE1-AA451-475 deleted). TN*wt*, TN*dl*IE1 and TN*rv*IE1 were described previously [49, 50]. TN-IE1_(Δ451–475)_ was generated by deleting the sequence encoding AA451-475 of IE1 from TN*wt* BAC via homologous recombination in *E*.*coli* DY380, followed by reconstitution in HELs as described previously [77, 78]. Viruses were propagated in HELs (HCMV, TN*wt* and TN*rv*IE1) or HELF-IE1 cells (TN*dl*IE1 and TN-IE1_(Δ451–475)_), titrated by standard plaque forming assay on monolayers of HELs, and stored as aliquots at -80°C for later use [79]. For infection, NPCs were infected with the indicated viruses at the specified multiplicity of infection (MOI), and the inoculum was replaced with culture medium consisting of half fresh GM and half CM at 3hpi [10]. Infected cells were collected at the indicated time points for further analysis.

### Transgene expression

Exogenous gene expression in 293T (1.5×10^6^ cells in 100-mm dishes) was accomplished using the specified amounts of the indicated plasmid DNA for transfection by Ca_3_(PO_4_)_2_ precipitation following a standard protocol as described previously [80]. For NPCs, nucleofection with plasmid or transduction using lentivirus were performed as described previously [5]. Nucleofection was performed using the Amaxa Mouse NSC Nucleofector Kit (Lonza) following the manufacturer’s instructions. Briefly, NPCs (5×10^6^) were mixed with 100μl Mesenchymal Stem Cell (MSC) Nucleofector Solution (82μl Nucleofector Solution with 18μl Supplement 1) containing specified amount of indicated plasmid DNA, transferred to a certified cuvette, and transfected using Nucleofector Program A-033. After nucleofection, NPCs were gently transferred to 100-mm dishes coated with poly-D-lysine, cultured in GM/CM mixture (1:1) for further culture and experiments. Lentiviruses were prepared by transfecting 293T cells (1.5×10^6^ cells in 100-mm dishes) using 15μg pCDH empty vector (serving as the vector control), pCDH-IE1, pLKO.1-shRNA-IE1 or pLKO.1-shRNA-scram together with plasmids pML-Δ8.9 (12μg) and pVSV-G (8μg) by Ca_3_(PO_4_)_2_ precipitation following a standard protocol as described previously [80, 81]. Following a medium change at 12hpt, the supernatant containing the lentiviruses were harvested at 72hpt, titrated on 293T cells by quantifying GFP-positive cells, and stored as aliquots at -80°C for further application. NPCs were transduced with the corresponding lentiviruses at an MOI of 1, and the expression of the transgene(s) was determined by immunoblotting.

### Immunoblotting (IB)

Cells were collected at the indicated time points with cell scrapers, rinsed with ice-cold PBS, pelleted by centrifugation, snap-frozen in liquid nitrogen and stored at -80°C until use. Cell lysates were prepared by lysing cell pellets in Cell Lysis Buffer (Beyotime). Protein concentration of cell lysates was measured using the BCA Protein Assay Kit (Beyotime) according to the manufacturer’s protocol as described previously [80]. Equal amounts of protein were separated by SDS-PAGE and transferred to PVDF membranes (Millipore). After sequential incubation with the primary antibodies against the indicated target proteins and corresponding horseradish peroxidase (HRP)-conjugated secondary antibodies, the membranes were incubated with SuperSignal West Femto Maximum Sensitivity Substrate or SuperSignal West Pico Chemiluminescent Substrate (Life Technologies). The chemiluminescent signals were detected using a FluorChem HD2 System (Alpha Innotech) and analyzed densitometrically using ImageJ (National Institutes of Health). For analyzing the cell lysates by IB, β-actin served as a loading control. Primary mouse monoclonal antibodies for the following proteins were used: HCMV IE1 (IgG1, Abcam, Cat# ab30924), HCMV IE2 (IgG1, Santa Cruse, Cat#SC69835), IE1/2 (IgG1, Virusys, Cat# p1215), pp65 (IgG1, Virusys, Cat# p1205), Hes1 (IgG1, Abcam, Cat# ab119776) and β-actin (IgG1, Sungene Biotech, Cat# KM9001). HRP-conjugated goat anti-mouse IgG (IgG, Abbkine, Cat# A21010) was used as secondary antibody.

### Cycloheximide treatment

To determine whether *de novo* viral protein synthesis is required for Hes1 downregulation, NPCs nucleofected with 0.25μg pCDH-Hes1 were pre-treated with 0.1mg/ml cycloheximide (CHX, Sigma-Aldrich) for 1 hour prior to HCMV or mock infection, and CHX treatment was maintained throughout the infection and post infection culture. Cells were collected at 12hpi, and protein levels of IE1 and Hes1 were determined by IB. The half-life of exogenous Hes1 protein in 293T cells were measured and calculated as previously described [82]. Briefly, 293T cells were transfected with 5μg pEYFP, pEYFP-IE1, or pEYFP-Δ451–475 together with 1μg pCDH-Hes1. CHX (0.1mg/ml) treatment started at 24hpt, and cells were collected at the indicated time points, Hes1 protein levels were determined by IB.

### Immunofluorescence assay (IFA)

NPCs grown on coverslips coated with poly-D-lysine were infected with HCMV at an MOI of 0.5, and mock infection was performed as a control. Coverslips were collected at the indicated time points post infection and processed for IFA as described previously [83]. Mouse monoclonal antibodies for HCMV IE1 (IgG2a, clone p63-27, made in Prof. Britt’s lab) and Hes1 (IgG1, Abcam, Cat# ab119776) were used as primary antibodies, and FITC-anti-mouse-IgG2a (Invitrogen, Cat# 11-4210-82) and TRITC-anti-mouse-IgG1 (Southern Biotechnology, Cat# 1070–03) were applied as secondary antibodies. Nuclei were counterstained with Hoechst 33342 (Life Technologies). Images were obtained using a two-photon microscopy with the UltraVIEW VoX 3D Live Cell Imaging System (Perkin Elmer).

### Protein purification

*E*. *coli* BL21 containing pET-Hes1, pET-Sp100A, pET-IE1 or pET-IE1_(Δ451–475)_ respectively was grown in Luria-Bertani medium containing kanamycin (50μg/ml, Amresco) at 37°C with vigorous shaking (220rpm). Isopropyl-β-D-1-thiogalactopyranoside (IPTG, 1.0mM, Sigma-Aldrich) was applied to induce target protein expression when the OD_600_ reached 0.6. Cells were collected after induction at the optimized temperature for the specific times: His-Hes1 at 37°C for 8h, His-IE1 and His-IE1_(Δ451–475)_ at 25°C for 8h, and His-Sp100A at 16°C for overnight. These His-tagged proteins were affinity-purified using the cOmplete His-Tag Purification Resin (Roche) following the manufacturer’s instruction. Briefly, the centrifugation pelleted cells were resuspended in Buffer A (50mM NaH_2_PO4, 300mM NaCl, pH 8.0), lysed by ultrasonication, centrifuged at 4°C to remove the cell debris, and loaded to Buffer A equilibrated resin columns (Bio-Rad). Following a 3 hours’ rotation at 4°C, the column was rinsed with Buffer A containing 20mM imidazole. Then, the protein was eluted from the resin using Buffer A containing the following concentration of imidazole: 400mM for His-Hes1, 600mM for His-Sp100A, 60mM for His-IE1 and 120mM for His-Δ451–475. For *in vitro* ubiquitination analysis without IP (described below), the eluted proteins were concentrated in Amicon Ultra-15 Centrifugal Filter Unit with Ultracel-10 membrane (Millipore) following the manufacturer’s protocol. The purity of the purified or concentrated proteins was examined by SDS-PAGE followed by Coomassie Brilliant Blue staining (Sigma-Aldrich).

### Protein interaction analysis

For the protein complex immunoprecipitation assay (IP), the centrifugation pelleted cells were re-suspended in Cell Lysis Buffer (Beyotime) and spun at 12,000g for 5min to remove debris. For the *in vitro* pull-down assay, 1μg of each purified protein or GST-Ubc5a protein (BostonBiochem) was mixed and incubated in a total volume of 300μl Buffer A. Cell lysate containing 2μg protein or the whole protein mixture was incubated with 1μg of the indicated antibody in a total volume of 300μl Buffer A. After thoroughly mixing by rotation overnight at 4°C, 60μl of protein A+G agarose beads (Beyotime) were added to the reaction and incubated with rotation for additional 3 hours at 4°C. The IP complex was pelleted by centrifugation at 2,500g for 5min, washed by Cell Lysis Buffer (Beyotime), re-suspended in protein loading buffer, subjected to SDS-PAGE, and immunoblotted (IB) for the indicated targets. For DNase and RNase treatment, 10,000 units of DNase I (Qiagen) and 10μg RNase (TianGEN) were applied to pre-treat the cell lysate for 1 hour prior to IP [84]. The applied antibodies included mouse monoclonal antibodies against Hes1 (IgG1, Abcam, Cat# ab119776), IE1 (IgG1, Abcam, Cat# ab30924), β-actin (IgG1, Sungene Biotech, Cat# KM9001), polyclonal rabbit antibodies against Sp100A (IgG, Abcam, Cat# ab167605). Secondary antibodies included HRP-conjugated goat anti-mouse IgG (IgG, Abbkine, Cat# A21010) and goat anti-rabbit IgG (IgG, Abbkine, Cat# A21020). Normal mouse IgG (Boster, Cat# BA1046) or normal rabbit IgG (cell signaling, Cat# 2729) were used as non-specific antibody controls.

### Ubiquitination assays

For *in vivo* ubiquitination analysis, 12.5μM MG132 (Sigma-Aldrich) was applied to the cells. HCMV-infected-NPCs were treated with MG132 starting at 3hpi, and harvested at 12hpi. For transduced NPCs or transfected 293T cells, MG132 was administered when the expressions of the target proteins were confirmed, and cells were treated for 12h. DMSO was applied as the solvent control. Cell lysates were subjected to Hes1- or Myc-directed IP followed by IB using a polyclonal rabbit antibody for ubiquitin.

For the *in vitro* ubiquitination assay, 4μg of each indicated purified protein was mixed with 2μg ubiquitin, 0.1μg E1 enzyme and 0.5μg Ubc5a (all from BostonBiochem) in a total volume of 1ml reaction buffer containing 50mM Tris-HCl (pH 7.4), 5mM MgCl_2_, 2mM DTT, 4mM ATP (Fermentas) and 20μM MG132 (Sigma-Aldrich) [85]. The mixture was incubated at 37°C or 4°C for 2 hours, and then quenched by protease inhibitor cocktail (Roche) and 1 mM N-ethylmaleimide (Sigma) on ice [86]. The cell lysate or reaction mixture was then subjected to the Hes1-, Sp100A-directed IP, and followed by IB against ubiquitin.

In addition, an *in vitro* ubiquitination assay was also performed without IP, with using double amount of each indicated purified protein, and the reaction mixture was directly subjected to IB with anti-Hes1. The ubiquitination reaction included 8μg of purified and concentrated proteins of His-IE1 or His-Δ451–475, and His-Hes1, 4μg ubiquitin, 0.5μg E1 enzyme and 1μg Ubc5a in 60μl reaction buffer as described above.

The primary antibodies included polyclonal rabbit anti-ubiquitin (IgG, ABclonal, Cat# A0162), -Sp100A (IgG, Abcam, Cat# ab167605) and -Ubc5a (IgG, Abcam, Cat# ab176561), monoclonal mouse anti-Hes1 (IgG1, Abcam, Cat# ab119776), -Myc (IgG1, Sigma, Cat# M4439), -β-actin (IgG1, Sungene Biotech, Cat# KM9001). The secondary antibodies included HRP-conjugated goat anti-mouse IgG (IgG, Abbkine, Cat# A21010) and goat anti-rabbit IgG (IgG, Abbkine, Cat# A21020).

### Statistical analysis

The immunoblotting and immunofluorescence images shown are representative of at least three independent experiments. The chemiluminescent signals of the immunoblotting images were densitometrically quantitated using ImageJ (National Institutes of Health). The relative levels of the target proteins were calculated as ratios to the corresponding control following β-actin normalization. The values are presented below the corresponding blot. The bar graphs were generated by using the GraphPad Prism software package based on the values from three independent experiments, and relative protein levels are represented as average ± standard deviation (SD). Differences were considered statistically significant when P≤0.05 based on a Student’s t-tests analysis. *, P ≤ 0.05; **, P ≤ 0.01.

## Supporting information

S1 Fig Downregulation of NICD1 and Jag1 proteins during HCMV infection in NPCs. Following mock (M)- or HCMV (V)-infection at an MOI of 3, NPCs were collected at the indicated times, and subjected to IB to examine IE1/2, NICD1, Jag1 and Hes1 proteins with β-actin as loading control. The values listed below the blots indicate the relative protein levels compared to corresponding mock controls following β-actin normalization. Representative results (A) and quantification (B) from 3 independent experiments are shown. *, P≤0.05; **, P≤0.01.
(TIF)

S2 Fig DNA and RNA are removed from the cell lysate by DNase/RNase treatment. Lysates of 293T cells were treated with 10,000 units DNase and 10μg RNase for 1h. DNA and RNA in non-treated and treated samples were visualized by ethidium bromide staining.
(TIF)

S3 Fig Interaction between Hes1 and IE1 or Δ421–475. (A) IE1/Hes1 interaction at different ratios. 293T cells were transfected with pCDH-Hes1 (Hes1) together with pEYFP-IE1 (IE1) or pEYFP (vector) at the indicated amounts. Cells were harvested at 48hpt and cell lysates were subjected to IE1-directed IP followed by IB against IE1 and Hes1. The proteins in the cell lysates were also examined. Normal IgG was used as the nonspecific antibody control. (B) IE1 but not Δ421–475 interacts with Hes1. 293T cells were transfected with 6μg of each indicated plasmid, including pCDH-Hes1 (Hes1), pEYFP-IE1 (IE1), pEYFP-IE1_(Δ421–475)_ (Δ421–475) and pEYFP (vector). Cells were harvested at 48hpt and cell lysates were subjected to Hes1-directed IP using antibody against Hes1 and subsequent IB for IE1 and Hes1. The proteins in the cell lysates were also examined. Normal IgG was used as the nonspecific antibody control.
(TIF)

S4 Fig IE1 but not Δ451–475 shortens the half-life of exogenous Hes1. (A) 293T cells were transfected with 1μg pCDH-Hes1 and 5μg pEYFP (vector), pEYFP-IE1 (IE1) or pEYFP-IE1_(Δ451–475)_ (Δ451–475). At 24hpt, cells were treated with 0.1mg/ml CHX and harvested at the indicated times post CHX treatment. The cell lysates were subjected to immunoblotting to examine Hes1 protein levels. To clearly visualize the protein signal of Hes1 in the presence of IE1, the exposure time was extended to 3 min for the IE1 expressing group compared to 30 seconds for others. (B) Hes1 degradation curve. Hes1 degradation curve was generated by taking log_2_ of the relative Hes1 protein levels (Hes1/β-actin) normalized to that of 0min (y) versus sampling time (x). The representative immunoblotting images (A) and average result (B) of 3 independent experiments are shown. The calculated formulas of the Hes1 degradation curves are as follows: vector, log_2_(*y*) = -0.0292*x*-0.1549, R^2^ = 0.9761; IE1, log_2_(*y*) = -0.0645*x*-0.0258, R^2^ = 0.9999; Δ451–475, log_2_(*y*) = -0.03*x*+0.0082, R^2^ = 0.9604. The Hes1 half-life (t_1/2_) is calculated according to the formulas as the *x* value when *y* is 0.5.
(TIF)

S5 Fig Prokaryotically expressed and purified proteins. His-Hes1, His-Δ451–475 and His-IE1 (A) or His-Sp100A and His-IE1 (B) were prokaryotically expressed, and purified as described in Materials and Methods. The indicated proteins in the according cell lysates, purified and/or concentrated protein samples were subjected to SDS-PAGE and subsequently stained with Coomassie Brilliant Blue.
(TIF)

S6 Fig Overexpressed Hes1 downregulates IE1/2 expression in HELs. Following transduction with lentiviruses expressing Hes1 (Hes1) or control (Ctl), HELs were infected with HCMV (MOI = 0.1) and examined for the protein levels of Hes1, IE1 and IE2 at the indicated time points by IB. β-actin served as a loading control. ND, not detectable.
(TIF)

## References

[1] Boppana SB, Pass RF, Britt WJ, Stagno S, Alford CA. Symptomatic Congenital Cytomegalovirus-Infection—Neonatal Morbidity and Mortality. Pediatr Infect Dis J. 1992;11(2):93–9. doi: 10.1097/00006454-199202000-00007 1311066 10.1097/00006454-199202000-00007

[2] Conboy TJ, Pass RF, Stagno S, Britt WJ, Alford CA, McFarland CE, et al Intellectual development in school-aged children with asymptomatic congenital cytomegalovirus infection. Pediatrics. 1986;77(6):801–6. . 3012452

[3] Rosenthal LS, Fowler KB, Boppana SB, Britt WJ, Pass RF, Schmid SD, et al Cytomegalovirus Shedding and Delayed Sensorineural Hearing Loss Results From Longitudinal Follow-up of Children With Congenital Infection. Pediatr Infect Dis J. 2009;28(6):515–20. doi: 10.1097/INF.0b013e318198c724 19483517 10.1097/INF.0b013e318198c724 PMC2757789

[4] Stagno S, Pass RF, Cloud G, Britt WJ, Henderson RE, Walton PD, et al Primary Cytomegalovirus-Infection in Pregnancy—Incidence, Transmission to Fetus, and Clinical Outcome. Jama-J Am Med Assoc. 1986;256(14):1904–8. 3020264

[5] Li XJ, Liu XJ, Yang B, Fu YR, Zhao F, Shen ZZ, et al Human Cytomegalovirus Infection Dysregulates the Localization and Stability of NICD1 and Jag1 in Neural Progenitor Cells. Journal of virology. 2015;89(13):6792–804. doi: 10.1128/JVI.00351-15 ; 25903338 10.1128/JVI.00351-15 PMC4468470

[6] Luo MH, Hannemann H, Kulkarni AS, Schwartz PH, O'Dowd JM, Fortunato EA. Human cytomegalovirus infection causes premature and abnormal differentiation of human neural progenitor cells. Journal of virology. 2010;84(7):3528–41. doi: 10.1128/JVI.02161-09 ; 20071566 10.1128/JVI.02161-09 PMC2838134

[7] Luo MH, Schwartz PH, Fortunato EA. Neonatal neural progenitor cells and their neuronal and glial cell derivatives are fully permissive for human cytomegalovirus infection. Journal of virology. 2008;82(20):9994–10007. doi: 10.1128/JVI.00943-08 ; 18684829 10.1128/JVI.00943-08 PMC2566273

[8] Odeberg J, Wolmer N, Falci S, Westgren M, Seiger A, Soderberg-Naucler C. Human cytomegalovirus inhibits neuronal differentiation and induces apoptosis in human neural precursor cells. Journal of virology. 2006;80(18):8929–39. doi: 10.1128/JVI.00676-06 16940505 10.1128/JVI.00676-06 PMC1563895

[9] Odeberg J, Wolmer N, Falci S, Westgren M, Sundtrom E, Seiger A, et al Late human cytomegalovirus (HCMV) proteins inhibit differentiation of human neural precursor cells into astrocytes. J Neurosci Res. 2007;85(3):583–93. doi: 10.1002/jnr.21144 17154414 10.1002/jnr.21144

[10] Pan X, Li XJ, Liu XJ, Yuan H, Li JF, Duan YL, et al Later passages of neural progenitor cells from neonatal brain are more permissive for human cytomegalovirus infection. Journal of virology. 2013;87(20):10968–79. doi: 10.1128/JVI.01120-13 ; 23903847 10.1128/JVI.01120-13 PMC3807278

[11] Gaiano N, Fishell G. The role of notch in promoting glial and neural stem cell fates. Annual review of neuroscience. 2002;25:471–90. doi: 10.1146/annurev.neuro.25.030702.130823 . 12052917 10.1146/annurev.neuro.25.030702.130823

[12] Selkoe D, Kopan R. Notch and Presenilin: regulated intramembrane proteolysis links development and degeneration. Annual review of neuroscience. 2003;26:565–97. doi: 10.1146/annurev.neuro.26.041002.131334 . 12730322 10.1146/annurev.neuro.26.041002.131334

[13] Shimojo H, Ohtsuka T, Kageyama R. Oscillations in notch signaling regulate maintenance of neural progenitors. Neuron. 2008;58(1):52–64. doi: 10.1016/j.neuron.2008.02.014 . 18400163 10.1016/j.neuron.2008.02.014

[14] Kageyama R, Ohtsuka T. The Notch-Hes pathway in mammalian neural development. Cell Res. 1999;9(3):179–88. doi: 10.1038/sj.cr.7290016 . 10520600 10.1038/sj.cr.7290016

[15] Borggrefe T, Oswald F. The Notch signaling pathway: transcriptional regulation at Notch target genes. Cell Mol Life Sci. 2009;66(10):1631–46. doi: 10.1007/s00018-009-8668-7 . 19165418 10.1007/s00018-009-8668-7 PMC11115614

[16] Kageyama R, Ohtsuka T, Kobayashi T. Roles of Hes genes in neural development. Dev Growth Differ. 2008;50:S97–S103. doi: 10.1111/j.1440-169X.2008.00993.x 18430159 10.1111/j.1440-169X.2008.00993.x

[17] Ohtsuka T, Sakamoto M, Guillemot F, Kageyama R. Roles of the basic helix-loop-helix genes Hes1 and Hes5 in expansion of neural stem cells of the developing brain. The Journal of biological chemistry. 2001;276(32):30467–74. doi: 10.1074/jbc.M102420200 . 11399758 10.1074/jbc.M102420200

[18] Dhanesh SB, Subashini C, James J. Hes1: the maestro in neurogenesis. Cell Mol Life Sci. 2016;73(21):4019–42. doi: 10.1007/s00018-016-2277-z . 27233500 10.1007/s00018-016-2277-z PMC11108451

[19] Kageyama R, Ohtsuka T, Shimojo H, Imayoshi I. Dynamic regulation of Notch signaling in neural progenitor cells. Current opinion in cell biology. 2009;21(6):733–40. doi: 10.1016/j.ceb.2009.08.009 . 19783418 10.1016/j.ceb.2009.08.009

[20] Kageyama R. Roles of Hes bHLH genes in neural development. J Neurochem. 2006;98:3-.16774582

[21] Hatakeyama J, Bessho Y, Katoh K, Ookawara S, Fujioka M, Guillemot F, et al Hes genes regulate size, shape and histogenesis of the nervous system by control of the timing of neural stem cell differentiation. Development. 2004;131(22):5539–50. doi: 10.1242/dev.01436 . 15496443 10.1242/dev.01436

[22] Ohtsuka T, Ishibashi M, Gradwohl G, Nakanishi S, Guillemot F, Kageyama R. Hes1 and Hes5 as Notch effectors in mammalian neuronal differentiation. Embo J. 1999;18(8):2196–207. doi: 10.1093/emboj/18.8.2196 10205173 10.1093/emboj/18.8.2196 PMC1171303

[23] Ishibashi M, Ang SL, Shiota K, Nakanishi S, Kageyama R, Guillemot F. Targeted disruption of mammalian hairy and Enhancer of split homolog-1 (HES-1) leads to up-regulation of neural helix-loop-helix factors, premature neurogenesis, and severe neural tube defects. Gene Dev. 1995;9(24):3136–48. doi: 10.1101/gad.9.24.3136 8543157 10.1101/gad.9.24.3136

[24] Cau E, Gradwohl G, Casarosa S, Kageyama R, Guillemot F. Hes genes regulate sequential stages of neurogenesis in the olfactory epithelium. Development. 2000;127(11):2323–32. 10804175 10.1242/dev.127.11.2323

[25] Liu XJ, Jiang X, Huang SN, Sun JY, Zhao F, Zeng WB, et al Human cytomegalovirus infection dysregulates neural progenitor cell fate by disrupting Hes1 rhythm and down-regulating its expression. Virologica Sinica. 2017;32(3):188–98. doi: 10.1007/s12250-017-3956-0 . 28451898 10.1007/s12250-017-3956-0 PMC6598910

[26] Torres L, Tang Q. Immediate-Early (IE) gene regulation of cytomegalovirus: IE1- and pp71-mediated viral strategies against cellular defenses. Virologica Sinica. 2014;29(6):343–52. doi: 10.1007/s12250-014-3532-9 ; 25501994 10.1007/s12250-014-3532-9 PMC4654928

[27] Stinski MF, Isomura H. Role of the cytomegalovirus major immediate early enhancer in acute infection and reactivation from latency. Medical microbiology and immunology. 2008;197(2):223–31. doi: 10.1007/s00430-007-0069-7 . 18097687 10.1007/s00430-007-0069-7

[28] Ahn JH, Hayward GS. The major immediate-early proteins IE1 and IE2 of human cytomegalovirus colocalize with and disrupt PML-associated nuclear bodies at very early times in infected permissive cells. Journal of virology. 1997;71(6):4599–613. ; 9151854 10.1128/jvi.71.6.4599-4613.1997 PMC191682

[29] Greaves RF, Mocarski ES. Defective growth correlates with reduced accumulation of a viral DNA replication protein after low-multiplicity infection by a human cytomegalovirus ie1 mutant. Journal of virology. 1998;72(1):366–79. ; 9420235 10.1128/jvi.72.1.366-379.1998 PMC109384

[30] Gawn JM, Greaves RF. Absence of IE1 p72 protein function during low-multiplicity infection by human cytomegalovirus results in a broad block to viral delayed-early gene expression. Journal of virology. 2002;76(9):4441–55. doi: 10.1128/JVI.76.9.4441-4455.2002 ; 11932411 10.1128/JVI.76.9.4441-4455.2002 PMC155072

[31] Luu P, Flores O. Binding of SP1 to the immediate-early protein-responsive element of the human cytomegalovirus DNA polymerase promoter. Journal of virology. 1997;71(9):6683–91. 9261391 10.1128/jvi.71.9.6683-6691.1997 PMC191947

[32] Malone CL, Vesole DH, Stinski MF. Transactivation of a Human Cytomegalovirus Early Promoter by Gene-Products from the Immediate-Early Gene Ie2 and Augmentation by Ie1—Mutational Analysis of the Viral-Proteins. Journal of virology. 1990;64(4):1498–506. 2157038 10.1128/jvi.64.4.1498-1506.1990 PMC249283

[33] Reeves M, Woodhall D, Compton T, Sinclair J. Human Cytomegalovirus IE72 Protein Interacts with the Transcriptional Repressor hDaxx To Regulate LUNA Gene Expression during Lytic Infection. Journal of virology. 2010;84(14):7185–94. doi: 10.1128/JVI.02231-09 20444888 10.1128/JVI.02231-09 PMC2898242

[34] Nevels M, Paulus C, Shenk T. Human cytomegalovirus immediate-early 1 protein facilitates viral replication by antagonizing histone deacetylation. Proceedings of the National Academy of Sciences of the United States of America. 2004;101(49):17234–9. doi: 10.1073/pnas.0407933101 ; 15572445 10.1073/pnas.0407933101 PMC535392

[35] Nevels M, Brune W, Shenk T. SUMOylation of the human cytomegalovirus 72-kilodalton IE1 protein facilitates expression of the 86-kilodalton IE2 protein and promotes viral replication. Journal of virology. 2004;78(14):7803–12. doi: 10.1128/JVI.78.14.7803-7812.2004 ; 15220454 10.1128/JVI.78.14.7803-7812.2004 PMC434104

[36] Knoblach T, Grandel B, Seiler J, Nevels M, Paulus C. Human cytomegalovirus IE1 protein elicits a type II interferon-like host cell response that depends on activated STAT1 but not interferon-gamma. PLoS pathogens. 2011;7(4):e1002016 doi: 10.1371/journal.ppat.1002016 ; 21533215 10.1371/journal.ppat.1002016 PMC3077363

[37] Hwang ES, Zhang Z, Cai H, Huang DY, Huong SM, Cha CY, et al Human cytomegalovirus IE1-72 protein interacts with p53 and inhibits p53-dependent transactivation by a mechanism different from that of IE2-86 protein. Journal of virology. 2009;83(23):12388–98. doi: 10.1128/JVI.00304-09 ; 19776115 10.1128/JVI.00304-09 PMC2786713

[38] Reitsma JM, Sato H, Nevels M, Terhune SS, Paulus C. Human cytomegalovirus IE1 protein disrupts interleukin-6 signaling by sequestering STAT3 in the nucleus. Journal of virology. 2013;87(19):10763–76. doi: 10.1128/JVI.01197-13 ; 23903834 10.1128/JVI.01197-13 PMC3807375

[39] Lee K, Jeon K, Kim JM, Kim VN, Choi DH, Kim SU, et al Downregulation of GFAP, TSP-1, and p53 in human glioblastoma cell line, U373MG, by IE1 protein from human cytomegalovirus. Glia. 2005;51(1):1–12. doi: 10.1002/glia.20179 . 15779089 10.1002/glia.20179

[40] Koh K, Lee K, Ahn JH, Kim S. Human cytomegalovirus infection downregulates the expression of glial fibrillary acidic protein in human glioblastoma U373MG cells: identification of viral genes and protein domains involved. The Journal of general virology. 2009;90(Pt 4):954–62. doi: 10.1099/vir.0.006486-0 . 19264642 10.1099/vir.0.006486-0

[41] Scherer M, Klingl S, Sevvana M, Otto V, Schilling EM, Stump JD, et al Crystal structure of cytomegalovirus IE1 protein reveals targeting of TRIM family member PML via coiled-coil interactions. PLoS pathogens. 2014;10(11):e1004512 doi: 10.1371/journal.ppat.1004512 ; 25412268 10.1371/journal.ppat.1004512 PMC4239116

[42] Li Y, Wu H, Wu W, Zhuo W, Liu W, Zhang Y, et al Structural insights into the TRIM family of ubiquitin E3 ligases. Cell Res. 2014;24(6):762–5. doi: 10.1038/cr.2014.46 ; 24722452 10.1038/cr.2014.46 PMC4042170

[43] Boutell C, Sadis S, Everett RD. Herpes simplex virus type 1 immediate-early protein ICP0 and is isolated RING finger domain act as ubiquitin E3 ligases in vitro. Journal of virology. 2002;76(2):841–50. doi: 10.1128/JVI.76.2.841-850.2002 ; 11752173 10.1128/JVI.76.2.841-850.2002 PMC136846

[44] Brulois K, Toth Z, Wong LY, Feng P, Gao SJ, Ensser A, et al Kaposi's sarcoma-associated herpesvirus K3 and K5 ubiquitin E3 ligases have stage-specific immune evasion roles during lytic replication. Journal of virology. 2014;88(16):9335–49. doi: 10.1128/JVI.00873-14 ; 24899205 10.1128/JVI.00873-14 PMC4136276

[45] Hagglund R, Van Sant C, Lopez P, Roizman B. Herpes simplex virus 1-infected cell protein 0 contains two E3 ubiquitin ligase sites specific for different E2 ubiquitin-conjugating enzymes. Proceedings of the National Academy of Sciences of the United States of America. 2002;99(2):631–6. doi: 10.1073/pnas.022531599 11805320 10.1073/pnas.022531599 PMC117357

[46] Moriuchi H, Moriuchi M, Smith HA, Straus SE, Cohen JI. Varicella-Zoster Virus Open Reading Frame 61 Protein Is Functionally Homologous to Herpes-Simplex Virus Type-1 Icpo. Journal of virology. 1992;66(12):7303–8. 1366099 10.1128/jvi.66.12.7303-7308.1992 PMC240434

[47] Walters MS, Kyratsous CA, Silverstein SJ. The RING Finger Domain of Varicella-Zoster Virus ORF61p Has E3 Ubiquitin Ligase Activity That Is Essential for Efficient Autoubiquitination and Dispersion of Sp100-Containing Nuclear Bodies. Journal of virology. 2010;84(13):6861–5. doi: 10.1128/JVI.00335-10 20392849 10.1128/JVI.00335-10 PMC2903287

[48] Yu Y, Wang SE, Hayward GS. The KSHV immediate-early transcription factor RTA encodes ubiquitin E3 ligase activity that targets IRF7 for proteosome-mediated degradation. Immunity. 2005;22(1):59–70. doi: 10.1016/j.immuni.2004.11.011 . 15664159 10.1016/j.immuni.2004.11.011

[49] Krauss S, Kaps J, Czech N, Paulus C, Nevels M. Physical requirements and functional consequences of complex formation between the cytomegalovirus IE1 protein and human STAT2. Journal of virology. 2009;83(24):12854–70. doi: 10.1128/JVI.01164-09 ; 19812155 10.1128/JVI.01164-09 PMC2786848

[50] Marchini A, Liu H, Zhu H. Human cytomegalovirus with IE-2 (UL122) deleted fails to express early lytic genes. Journal of virology. 2001;75(4):1870–8. doi: 10.1128/JVI.75.4.1870-1878.2001 ; 11160686 10.1128/JVI.75.4.1870-1878.2001 PMC114097

[51] Mucke K, Paulus C, Bernhardt K, Gerrer K, Schon K, Fink A, et al Human cytomegalovirus major immediate early 1 protein targets host chromosomes by docking to the acidic pocket on the nucleosome surface. Journal of virology. 2014;88(2):1228–48. doi: 10.1128/JVI.02606-13 ; 24227840 10.1128/JVI.02606-13 PMC3911650

[52] Hirata H, Yoshiura S, Ohtsuka T, Bessho Y, Harada T, Yoshikawa K, et al Oscillatory expression of the bHLH factor Hes1 regulated by a negative feedback loop. Science. 2002;298(5594):840–3. doi: 10.1126/science.1074560 . 12399594 10.1126/science.1074560

[53] Huh YH, Kim YE, Kim ET, Park JJ, Song MJ, Zhu H, et al Binding STAT2 by the acidic domain of human cytomegalovirus IE1 promotes viral growth and is negatively regulated by SUMO. Journal of virology. 2008;82(21):10444–54. doi: 10.1128/JVI.00833-08 ; 18701593 10.1128/JVI.00833-08 PMC2573188

[54] Calistri A, Munegato D, Carli I, Parolin C, Palu G. The ubiquitin-conjugating system: multiple roles in viral replication and infection. Cells. 2014;3(2):386–417. doi: 10.3390/cells3020386 ; 24805990 10.3390/cells3020386 PMC4092849

[55] Gu H, Roizman B. The degradation of promyelocytic leukemia and Sp100 proteins by herpes simplex virus 1 is mediated by the ubiquitin-conjugating enzyme UbcH5a. Proceedings of the National Academy of Sciences of the United States of America. 2003;100(15):8963–8. doi: 10.1073/pnas.1533420100 ; 12855769 10.1073/pnas.1533420100 PMC166421

[56] Pickart CM, Eddins MJ. Ubiquitin: structures, functions, mechanisms. Biochimica et biophysica acta. 2004;1695(1–3):55–72. doi: 10.1016/j.bbamcr.2004.09.019 . 15571809 10.1016/j.bbamcr.2004.09.019

[57] Zhao Q, Liang D, Sun R, Jia B, Xia T, Xiao H, et al Kaposi's sarcoma-associated herpesvirus-encoded replication and transcription activator impairs innate immunity via ubiquitin-mediated degradation of myeloid differentiation factor 88. Journal of virology. 2015;89(1):415–27. doi: 10.1128/JVI.02591-14 ; 25320320 10.1128/JVI.02591-14 PMC4301122

[58] Kim YE, Lee JH, Kim ET, Shin HJ, Gu SY, Seol HS, et al Human cytomegalovirus infection causes degradation of Sp100 proteins that suppress viral gene expression. Journal of virology. 2011;85(22):11928–37. doi: 10.1128/JVI.00758-11 ; 21880768 10.1128/JVI.00758-11 PMC3209270

[59] Tavalai N, Adler M, Scherer M, Riedl Y, Stamminger T. Evidence for a dual antiviral role of the major nuclear domain 10 component Sp100 during the immediate-early and late phases of the human cytomegalovirus replication cycle. Journal of virology. 2011;85(18):9447–58. doi: 10.1128/JVI.00870-11 ; 21734036 10.1128/JVI.00870-11 PMC3165758

[60] Figueiredo-Pereira ME, Rockwell P, Schmidt-Glenewinkel T, Serrano P. Neuroinflammation and J2 prostaglandins: linking impairment of the ubiquitin-proteasome pathway and mitochondria to neurodegeneration. Frontiers in molecular neuroscience. 2014;7:104 doi: 10.3389/fnmol.2014.00104 ; 25628533 10.3389/fnmol.2014.00104 PMC4292445

[61] Yi JJ, Ehlers MD. Emerging roles for ubiquitin and protein degradation in neuronal function. Pharmacological reviews. 2007;59(1):14–39. doi: 10.1124/pr.59.1.4 . 17329546 10.1124/pr.59.1.4

[62] Loureiro J, Ploegh HL. Antigen presentation and the ubiquitin-proteasome system in host-pathogen interactions. Advances in immunology. 2006;92:225–305. doi: 10.1016/S0065-2776(06)92006-9 . 17145306 10.1016/S0065-2776(06)92006-9 PMC7112114

[63] Blanchette P, Branton PE. Manipulation of the ubiquitin-proteasome pathway by small DNA tumor viruses. Virology. 2009;384(2):317–23. doi: 10.1016/j.virol.2008.10.005 19013629 10.1016/j.virol.2008.10.005

[64] Petroski MD. The ubiquitin system, disease, and drug discovery. Bmc Biochem. 2008;9 Artn S7 doi: 10.1186/1471-2091-9-S1-S7 19007437 10.1186/1471-2091-9-S1-S7 PMC2582801

[65] Delboy MG, Roller DG, Nicola AV. Cellular proteasome activity facilitates herpes simplex virus entry at a postpenetration step. Journal of virology. 2008;82(7):3381–90. doi: 10.1128/JVI.02296-07 18234803 10.1128/JVI.02296-07 PMC2268500

[66] Greene W, Zhang W, He ML, Witt C, Ye FC, Gao SJ. The Ubiquitin/Proteasome System Mediates Entry and Endosomal Trafficking of Kaposi's Sarcoma-Associated Herpesvirus in Endothelial Cells. PLoS pathogens. 2012;8(5). ARTN e1002703. doi: 10.1371/journal.ppat.1002703 22615563 10.1371/journal.ppat.1002703 PMC3355089

[67] Tran K, Mahr JA, Spector DH. Proteasome Subunits Relocalize during Human Cytomegalovirus Infection, and Proteasome Activity Is Necessary for Efficient Viral Gene Transcription. Journal of virology. 2010;84(6):3079–93. doi: 10.1128/JVI.02236-09 20042513 10.1128/JVI.02236-09 PMC2826056

[68] Hershko A, Ciechanover A. The ubiquitin system. Annual review of biochemistry. 1998;67:425–79. doi: 10.1146/annurev.biochem.67.1.425 . 9759494 10.1146/annurev.biochem.67.1.425

[69] Metzger MB, Pruneda JN, Klevit RE, Weissman AM. RING-type E3 ligases: master manipulators of E2 ubiquitin-conjugating enzymes and ubiquitination. Biochimica et biophysica acta. 2014;1843(1):47–60. doi: 10.1016/j.bbamcr.2013.05.026 ; 23747565 10.1016/j.bbamcr.2013.05.026 PMC4109693

[70] Deshaies RJ, Joazeiro CA. RING domain E3 ubiquitin ligases. Annual review of biochemistry. 2009;78:399–434. doi: 10.1146/annurev.biochem.78.101807.093809 . 19489725 10.1146/annurev.biochem.78.101807.093809

[71] Randow F, Lehner PJ. Viral avoidance and exploitation of the ubiquitin system. Nature cell biology. 2009;11(5):527–34. doi: 10.1038/ncb0509-527 . 19404332 10.1038/ncb0509-527

[72] Ahn JH, Brignole EJ 3rd, Hayward GS. Disruption of PML subnuclear domains by the acidic IE1 protein of human cytomegalovirus is mediated through interaction with PML and may modulate a RING finger-dependent cryptic transactivator function of PML. Molecular and cellular biology. 1998;18(8):4899–913. ; 9671498 10.1128/mcb.18.8.4899 PMC109074

[73] Ardley HC, Robinson PA. E3 ubiquitin ligases. Essays in biochemistry. 2005;41:15–30. doi: 10.1042/EB0410015 . 16250895 10.1042/EB0410015

[74] Bijlmakers MJ, Teixeira JM, Boer R, Mayzel M, Puig-Sarries P, Karlsson G, et al A C2HC zinc finger is essential for the RING-E2 interaction of the ubiquitin ligase RNF125. Sci Rep. 2016;6:29232 doi: 10.1038/srep29232 ; 27411375 10.1038/srep29232 PMC4944129

[75] Reuter N, Schilling EM, Scherer M, Muller R, Stamminger T. The ND10 component promyelocytic leukemia protein acts as an E3 ligase for SUMOylation of the major immediate-early protein IE1 of human cytomegalovirus. Journal of virology. 2017 doi: 10.1128/JVI.02335-16 . 28250117 10.1128/JVI.02335-16 PMC5411614

[76] Wagenknecht N, Reuter N, Scherer M, Reichel A, Muller R, Stamminger T. Contribution of the Major ND10 Proteins PML, hDaxx and Sp100 to the Regulation of Human Cytomegalovirus Latency and Lytic Replication in the Monocytic Cell Line THP-1. Viruses. 2015;7(6):2884–907. doi: 10.3390/v7062751 ; 26057166 10.3390/v7062751 PMC4488718

[77] Zhao F, Shen ZZ, Liu ZY, Zeng WB, Cheng S, Ma YP, et al Identification and BAC construction of Han, the first characterized HCMV clinical strain in China. Journal of medical virology. 2016;88(5):859–70. doi: 10.1002/jmv.24396 . 26426373 10.1002/jmv.24396

[78] Zhang Z, Huang Y, Zhu H. A highly efficient protocol of generating and analyzing VZV ORF deletion mutants based on a newly developed luciferase VZV BAC system. Journal of virological methods. 2008;148(1–2):197–204. doi: 10.1016/j.jviromet.2007.11.012 ; 18215429 10.1016/j.jviromet.2007.11.012 PMC2291443

[79] Casavant NC, Luo MH, Rosenke K, Winegardner T, Zurawska A, Fortunato EA. Potential role for p53 in the permissive life cycle of human cytomegalovirus. Journal of virology. 2006;80(17):8390–401. doi: 10.1128/JVI.00505-06 ; 16912290 10.1128/JVI.00505-06 PMC1563868

[80] Fu YR, Liu XJ, Li XJ, Shen ZZ, Yang B, Wu CC, et al MicroRNA miR-21 attenuates human cytomegalovirus replication in neural cells by targeting Cdc25a. Journal of virology. 2015;89(2):1070–82. doi: 10.1128/JVI.01740-14 ; 25378484 10.1128/JVI.01740-14 PMC4300626

[81] Tiscornia G, Singer O, Verma IM. Production and purification of lentiviral vectors. Nature protocols. 2006;1(1):241–5. doi: 10.1038/nprot.2006.37 . 17406239 10.1038/nprot.2006.37

[82] Ajiro M, Zheng ZM. E6^E7, a novel splice isoform protein of human papillomavirus 16, stabilizes viral E6 and E7 oncoproteins via HSP90 and GRP78. mBio. 2015;6(1):e02068–14. doi: 10.1128/mBio.02068-14 ; 25691589 10.1128/mBio.02068-14 PMC4337564

[83] Duan Y, Miao L, Ye H, Yang C, Fu B, Schwartz PH, et al A faster immunofluorescence assay for tracking infection progress of human cytomegalovirus. Acta biochimica et biophysica Sinica. 2012;44(7):597–605. doi: 10.1093/abbs/gms041 ; 22659494 10.1093/abbs/gms041 PMC3382298

[84] Ho SR, Mahanic CS, Lee YJ, Lin WC. RNF144A, an E3 ubiquitin ligase for DNA-PKcs, promotes apoptosis during DNA damage. Proceedings of the National Academy of Sciences of the United States of America. 2014;111(26):E2646–55. doi: 10.1073/pnas.1323107111 ; 24979766 10.1073/pnas.1323107111 PMC4084471

[85] Huang F, Xiao H, Sun BL, Yang RG. Characterization of TRIM62 as a RING finger E3 ubiquitin ligase and its subcellular localization. Biochemical and biophysical research communications. 2013;432(2):208–13. doi: 10.1016/j.bbrc.2013.02.012 . 23402750 10.1016/j.bbrc.2013.02.012

[86] Liu Y, Fallon L, Lashuel HA, Liu Z, Lansbury PT Jr. The UCH-L1 gene encodes two opposing enzymatic activities that affect alpha-synuclein degradation and Parkinson's disease susceptibility. Cell. 2002;111(2):209–18. . 12408865 10.1016/s0092-8674(02)01012-7

