# Supplementary figures and images for: Human cytomegalovirus IE1 downregulates Hes1 in neural progenitor cells as a potential E3 ubiquitin ligase
Source: PLoS Pathog. 2017 Jul 27;13(7):e1006542. doi: 10.1371/journal.ppat.1006542 (PMC5549770; doi:10.1371/journal.ppat.1006542)

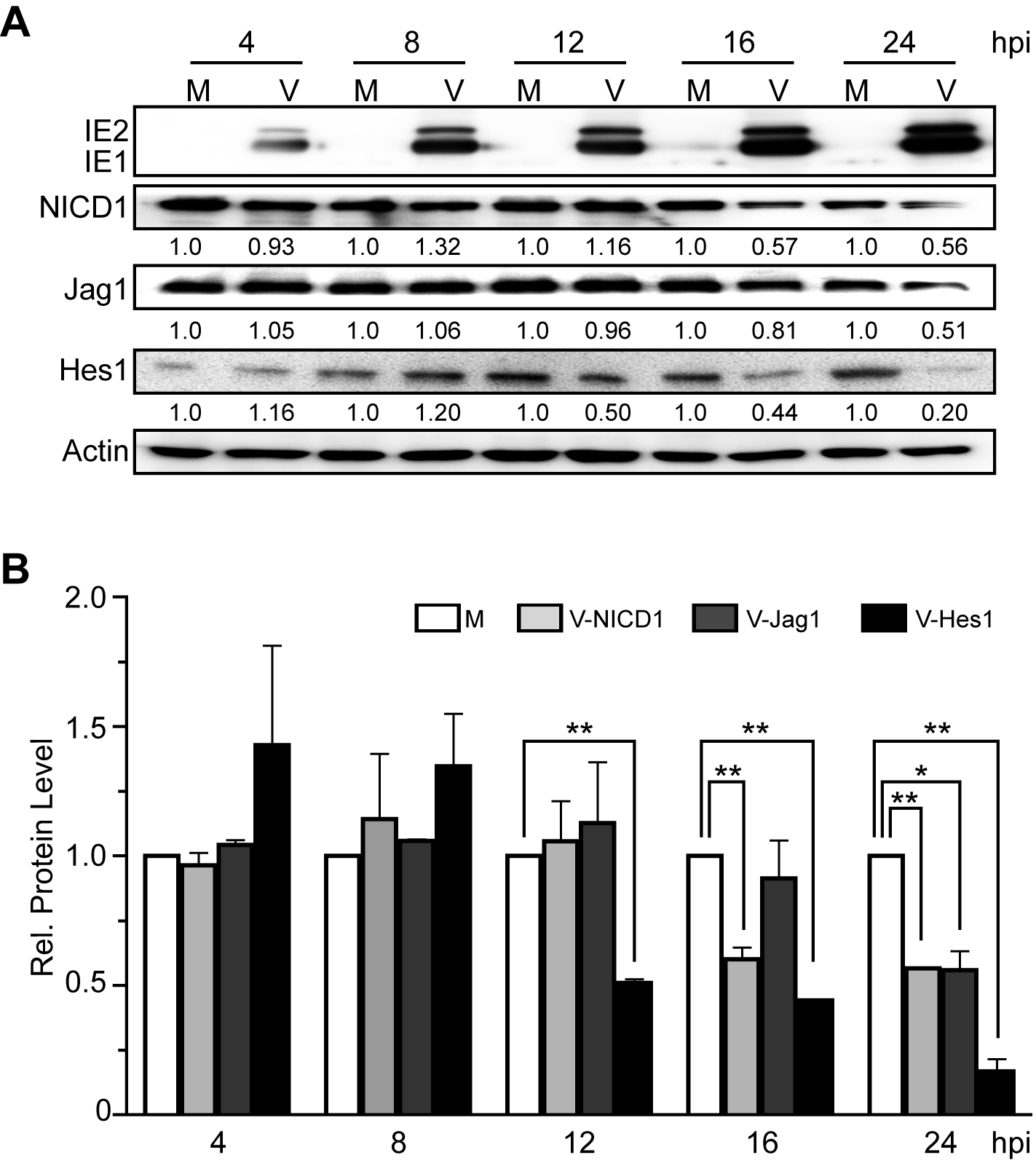

Supplement: S1 Fig — Following mock (M)- or HCMV (V)-infection at an MOI of 3, NPCs were collected at the indicated times, and subjected to IB to examine IE1/2, NICD1, Jag1 and Hes1 proteins with β-actin as loading control. The values listed below the blots indicate the relative protein levels compared to corresponding mock controls following β-actin normalization. Representative results (A) and quantification (B) from 3 independent experiments are shown. *, P≤0.05; **, P≤0.01. (TIF) [file ppat.1006542.s001.tif]

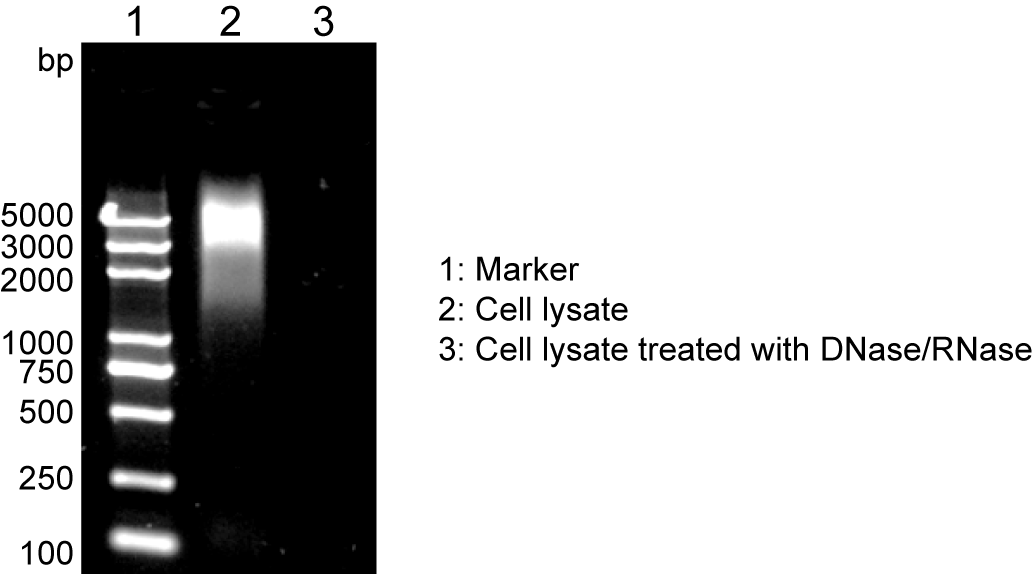

Supplement: S2 Fig — Lysates of 293T cells were treated with 10,000 units DNase and 10μg RNase for 1h. DNA and RNA in non-treated and treated samples were visualized by ethidium bromide staining. (TIF) [file ppat.1006542.s002.tif]

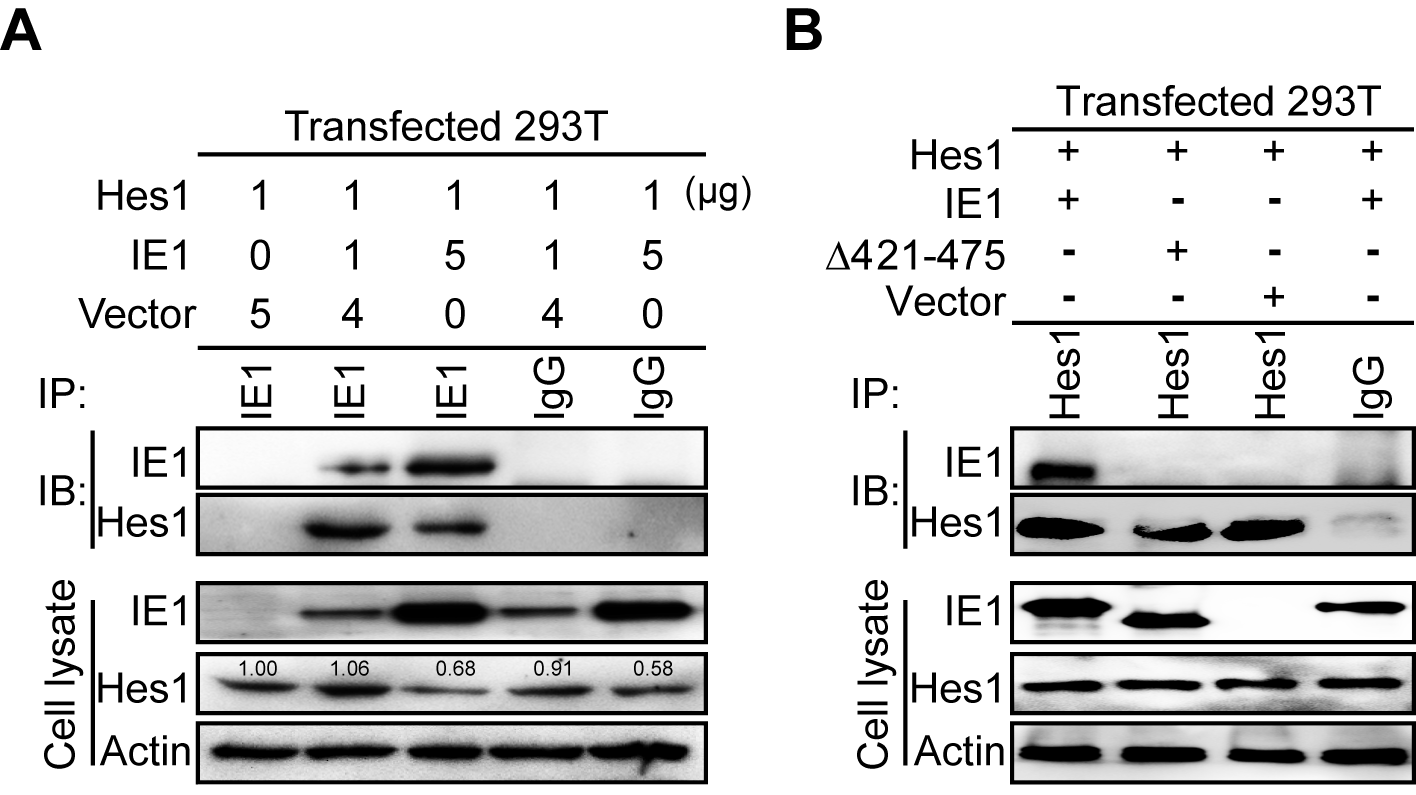

Supplement: S3 Fig — (A) IE1/Hes1 interaction at different ratios. 293T cells were transfected with pCDH-Hes1 (Hes1) together with pEYFP-IE1 (IE1) or pEYFP (vector) at the indicated amounts. Cells were harvested at 48hpt and cell lysates were subjected to IE1-directed IP followed by IB against IE1 and Hes1. The proteins in the cell lysates were also examined. Normal IgG was used as the nonspecific antibody control. (B) IE1 but not Δ421–475 interacts with Hes1. 293T cells were transfected with 6μg of each indicated plasmid, including pCDH-Hes1 (Hes1), pEYFP-IE1 (IE1), pEYFP-IE1(Δ421–475) (Δ421–475) and pEYFP (vector). Cells were harvested at 48hpt and cell lysates were subjected to Hes1-directed IP using antibody against Hes1 and subsequent IB for IE1 and Hes1. The proteins in the cell lysates were also examined. Normal IgG was used as the nonspecific antibody control. (TIF) [file ppat.1006542.s003.tif]

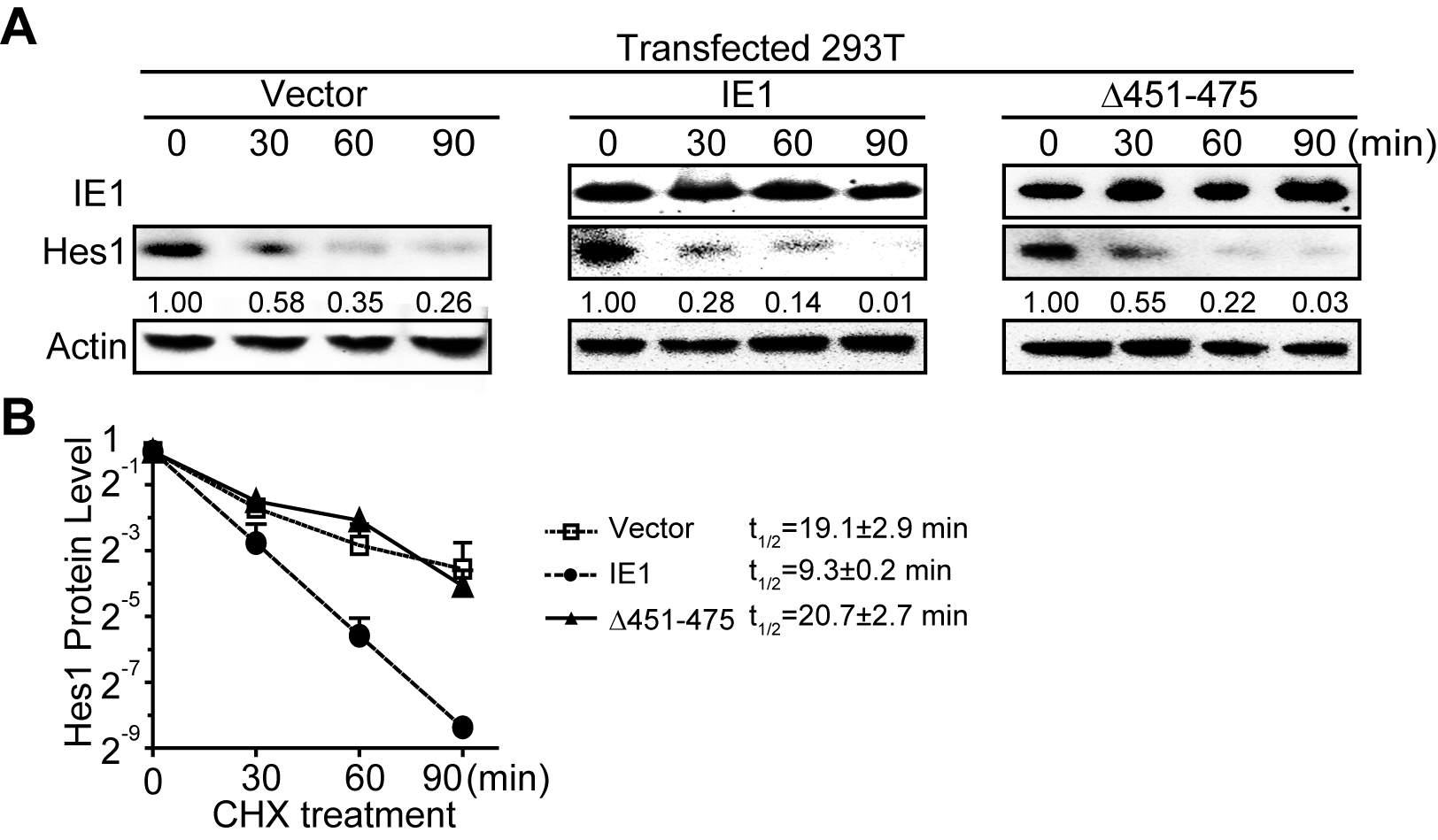

Supplement: S4 Fig — (A) 293T cells were transfected with 1μg pCDH-Hes1 and 5μg pEYFP (vector), pEYFP-IE1 (IE1) or pEYFP-IE1(Δ451–475) (Δ451–475). At 24hpt, cells were treated with 0.1mg/ml CHX and harvested at the indicated times post CHX treatment. The cell lysates were subjected to immunoblotting to examine Hes1 protein levels. To clearly visualize the protein signal of Hes1 in the presence of IE1, the exposure time was extended to 3 min for the IE1 expressing group compared to 30 seconds for others. (B) Hes1 degradation curve. Hes1 degradation curve was generated by taking log2 of the relative Hes1 protein levels (Hes1/β-actin) normalized to that of 0min (y) versus sampling time (x). The representative immunoblotting images (A) and average result (B) of 3 independent experiments are shown. The calculated formulas of the Hes1 degradation curves are as follows: vector, log2(y) = -0.0292x-0.1549, R2 = 0.9761; IE1, log2(y) = -0.0645x-0.0258, R2 = 0.9999; Δ451–475, log2(y) = -0.03x+0.0082, R2 = 0.9604. The Hes1 half-life (t1/2) is calculated according to the formulas as the x value when y is 0.5. (TIF) [file ppat.1006542.s004.tif]

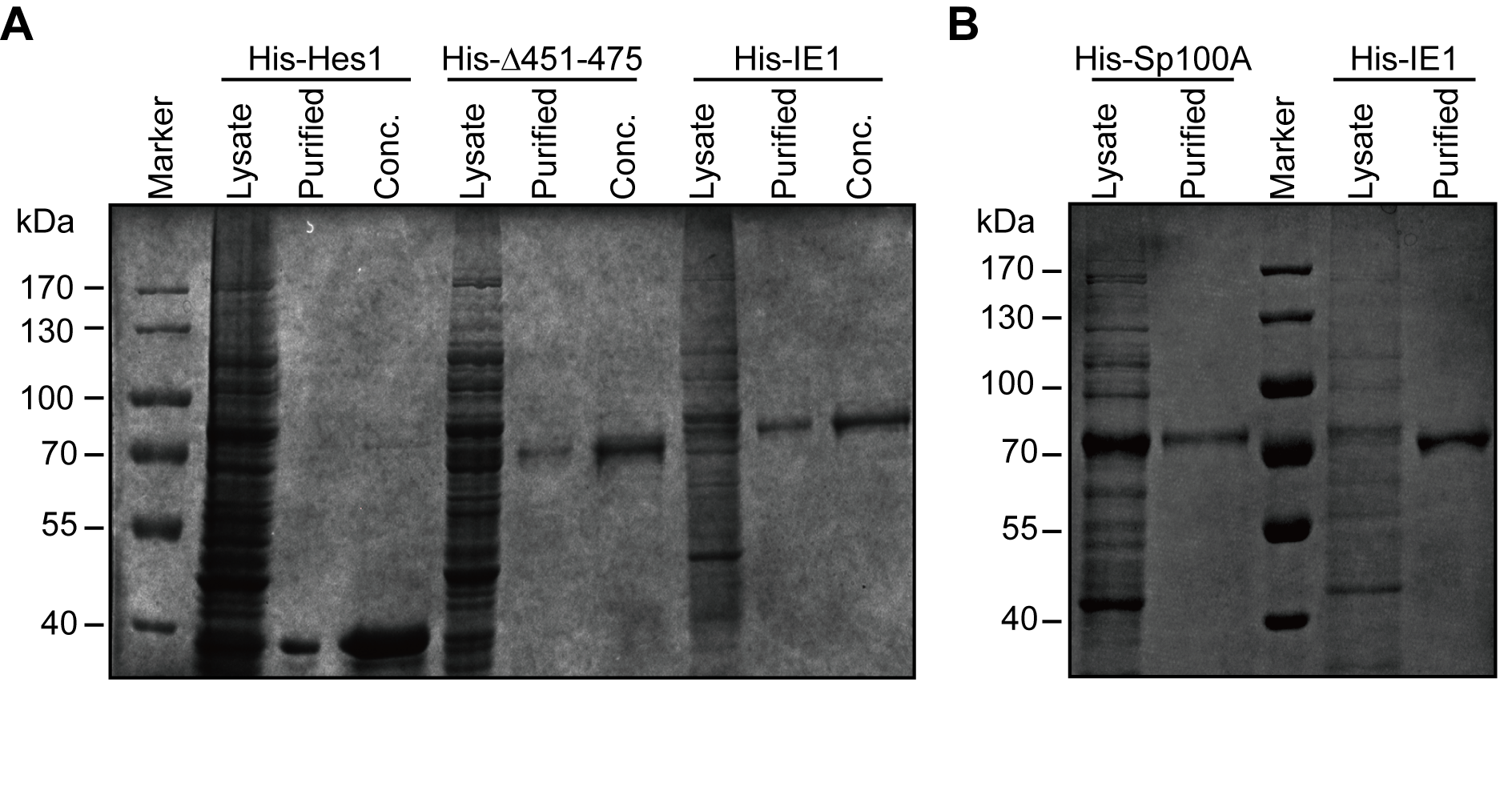

Supplement: S5 Fig — His-Hes1, His-Δ451–475 and His-IE1 (A) or His-Sp100A and His-IE1 (B) were prokaryotically expressed, and purified as described in Materials and Methods. The indicated proteins in the according cell lysates, purified and/or concentrated protein samples were subjected to SDS-PAGE and subsequently stained with Coomassie Brilliant Blue. (TIF) [file ppat.1006542.s005.tif]

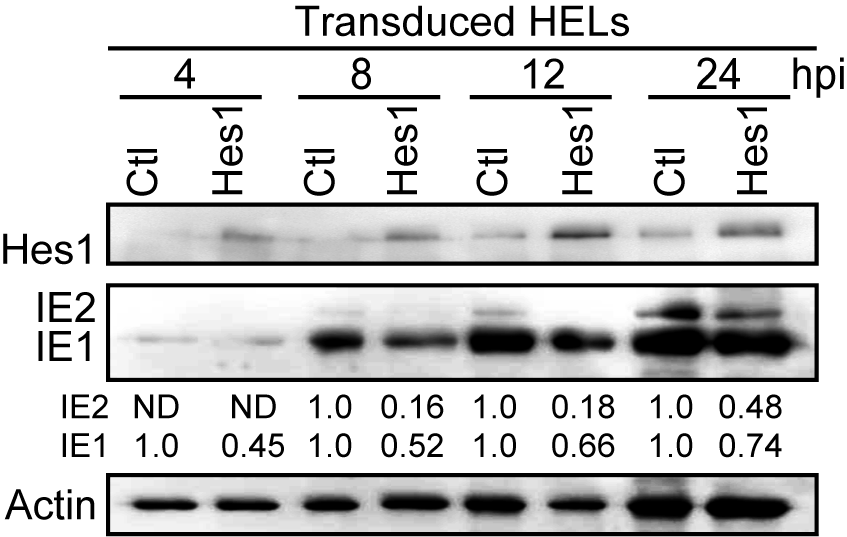

Supplement: S6 Fig — Following transduction with lentiviruses expressing Hes1 (Hes1) or control (Ctl), HELs were infected with HCMV (MOI = 0.1) and examined for the protein levels of Hes1, IE1 and IE2 at the indicated time points by IB. β-actin served as a loading control. ND, not detectable. (TIF) [file ppat.1006542.s006.tif]
